# A bibliometric analysis of drug resistance in immunotherapy for breast cancer: trends, themes, and research focus

**DOI:** 10.3389/fimmu.2024.1452303

**Published:** 2024-08-12

**Authors:** Rendong Zhang, Qiongzhi Jiang, Zhemin Zhuang, Huancheng Zeng, Yaochen Li

**Affiliations:** ^1^ Department of Breast Surgery, Cancer Hospital of Shantou University Medical College, Shantou, China; ^2^ Department of Radiation Oncology, Cancer Hospital of Shantou University Medical College, Shantou, China; ^3^ Engineering College, Shantou University, Shantou, Guangdong, China; ^4^ The Central Laboratory, Cancer Hospital of Shantou University Medical College, Shantou, China

**Keywords:** immunotherapy resistance, breast cancer, bibliometric analysis, VOSviewer, CiteSpace, mechanisms, tumor microenvironment

## Abstract

While breast cancer treatments have advanced significantly nowadays, yet metastatic, especially triple-negative breast cancer (TNBC), remains challenging with low survival. Cancer immunotherapy, a promising approach for HER2-positive and TNBC, still faces resistance hurdles. Recently, numerous studies have set their sights on the resistance of immunotherapy for breast cancer. Our study provides a thorough comprehension of the current research landscape, hotspots, and emerging breakthroughs in this critical area through a meticulous bibliometric analysis. As of March 26, 2024, a total of 1341 articles on immunology resistance in breast cancer have been gathered from Web of Science Core Collection, including 765 articles and 576 reviews. Bibliometrix, CiteSpace and VOSviewer software were utilized to examine publications and citations per year, prolific countries, contributive institutions, high-level journals and scholars, as well as highly cited articles, references and keywords. The research of immunotherapy resistance in breast cancer has witnessed a remarkable surge over the past seven years. The United States and China have made significant contributions, with Harvard Medical School being the most prolific institution and actively engaging in collaborations. The most contributive author is Curigliano, G from the European Institute of Oncology in Italy, while Wucherpfennig, K. W. from the Dana-Farber Cancer Institute in the USA, had the highest citations. Journals highly productive primarily focus on clinical, immunology and oncology research. Common keywords include “resistance”, “expression”, “tumor microenvironment”, “cancer”, “T cell”, “therapy”, “chemotherapy” and “cell”. Current research endeavors to unravel the mechanisms of immune resistance in breast cancer through the integration of bioinformatics, basic experiments, and clinical trials. Efforts are underway to develop strategies that improve the effectiveness of immunotherapy, including the exploration of combination therapies and advancements in drug delivery systems. Additionally, there is a strong focus on identifying novel biomarkers that can predict patient response to immunology. This study will provide researchers with an up-to-date overview of the present knowledge in drug resistance of immunology for breast cancer, serving as a valuable resource for informed decision-making and further research on innovative approaches to address immunotherapy resistance.

## 1 Introduction

In 2022, breast cancer continues to be the leading type of cancer affecting women worldwide as indicated by the latest GLOBOCAN estimates from International Agency for Research on Cancer (IARC) with 2,308,897 new cases worldwide, representing 11.6% of all female cancer cases, resulting in 665,684 deaths, accounting for 6.9% of all cancer-related fatalities, as the fourth most common cause of cancer deaths among women globally (1). For non-metastatic breast cancer, traditional treatment strategies encompass radical mastectomy or breast-conserving surgery for tumour removal, complemented by preoperative neoadjuvant or postoperative systemic therapies (2). Since 2000, breast cancer has been categorized into distinct molecular types based on the expression levels of estrogen receptor (ER), progesterone receptor (PR) and human epidermal growth factor receptor 2 (HER2), which is ER-positive/luminal-like, Erb-B2 positive, basal-like, normal breast-like and claudin-low, paving the way for personalized, effective and safe treatments (3–6). Targeted drugs, such as endocrine therapy for hormone receptor-positive breast cancer and anti-HER2 therapy for HER2-positive breast cancer have come to the fore (7, 8). Before the advent of HER2-targeted therapies, HER2-enriched breast cancer, which accounts for 10 to 15% of breast cancers, had the lowest 5-year survival rate among all subtypes (9). These advancements have significantly improved breast cancer prognosis, leading to an encouraging 5-year survival rate of 90.3% overall. However, for metastatic breast cancer, the 5-year survival rate significantly declines to 29% and further plummets to 12% for metastatic triple-negative breast cancer (TNBC) (10). Representing around 20% of breast cancers, TNBC is recognized as the most aggressive one now and has the bleakest prognosis owing to the absence of precisely targeted therapy options and a higher susceptibility to developing drug resistance to conventional treatments (11–13). In spite of the availability of diverse treatment modalities, the recurrence and metastasis of breast cancer remain formidable challenges for patient survival (14–17). Additionally, the development of resistance to chemotherapy, endocrine therapy and targeted therapy in certain individuals poses a significant obstacle as well (18–20).

In recent years, cancer immunotherapy, involving passive, active and adoptive immunotherapy, has come into view as a cutting-edge anti-tumor strategy (21). While most breast cancers were traditionally considered immunologically “cold” tumors with limited responsiveness to immunotherapy, TNBC, the most immunogenic subtype, demonstrates a notable response (22). Harnessing immune checkpoint inhibitors to target the PD-1/PD-L1 axis has exhibited noteworthy anti-tumor ability in TNBC patients, leading to potential long-term survival benefits and improved prognosis (23). Monotherapy studies with immune checkpoint inhibitors such as pembrolizumab and atezolizumab in metastatic TNBC have yielded objective response rates (ORRs) ranging from 4% to 23% (24, 25). Moreover, some HER2-positive breast cancer patients may also be eligible for immunotherapy (26). Combining immunotherapy with HER2-targeted therapy has emerged as a promising approach, particularly in patients with PD-L1 positivity and/or high levels of tumor infiltrating lymphocytes (TILs). For instance, the combination of pembrolizumab and trastuzumab has shown objective responses in HER2-positive and PD-L1-positive metastatic breast cancer patients who experienced progression on trastuzumab treatment (27). Furthermore, adding atezolizumab to T-DM1 for patients with locally advanced or metastatic HER2-positive breast cancer who had previously undergone treatment with trastuzumab and paclitaxel-based therapy, led to improved progression-free survival (PFS) in PD-L1-positive patients exhibiting TILs of at least 5% (28). Despite significant advancements in immunotherapy, it is crucial to recognize that many breast cancer patients still have limited response and are susceptible to disease progression or recurrence, known as immunotherapy resistance (29). The immunosuppressive tumor microenvironment poses a significant challenge to the effectiveness of immunotherapy, especially in aggressive breast cancer subtypes like HER2-positive and TNBC (30). Understanding the specific mechanisms that drive breast cancer immunotherapy resistance is essential for enhancing treatment efficacy.

Bibliometric analysis is an invaluable research methodology that utilizes statistical methods and visualization tools to quantify and interpret scholarly publications, providing researchers with a comprehensive understanding of the research landscape and trends within a specific field over a defined period (31). Despite the extensive exploration of immunotherapy resistance in breast cancer in numerous studies, there is currently a dearth of bibliometric analyses that systematically sort and examine the prevailing research trends and hotspots within this domain. To bridge this gap, we carried out a meticulous bibliometric analysis of documents on immunotherapy resistance in breast cancer published on Web of Science database in 2003-2024 using relevant scientometric softwares such as Bibliometrix, CiteSpace, and VOSviewer to analyze the annual publications output, productive countries, institutions, journals and authors involved in the field. Cooperative networks and knowledge graphs were constructed to visualize the results. Additionally, citation analysis of publications, keyword co-occurrence analysis and identification of citation bursts were performed to identify significant concerns of scholars. The objective of this study is to present the development context of drug resistance in immunotherapy for breast cancer, clarify the research trend and grasp the hot spots and research gaps to carry out follow-up comprehensive and in-depth research.

## 2 Materials and methods

### 2.1 Data collection and search strategy

Using editions including SCI-EXPANDED, SSCI, AHCI, ESCI, CCR-EXPANDED and IC of Web of Science Core Collection, the research was undertaken on March 26, 2024, to retrieve documents related to drug resistance in immunotherapy for breast cancer in the period from 2003 to the date of searching. The MeSh terms and entry terms of “breast cancer” and “immunotherapy resistance” were captured as search strategies, which are following: #1, TS=(“breast cancer$”) OR TS=(“breast carcinoma$”) OR TS=(“mammary cancer$”) OR TS=(“mammary carcinoma$”) OR TS=(“breast malignant tumor$”) OR TS=(“breast malignant neoplasm$”); #2, TS=(Immunotherap*);#3, TS=(resistan*); #4, “#1” and “#2” and “#3”. “TS” represents topic and “*” represents 0 or more characters. Primarily, a result of 2084 documents was retrieved. The inclusion criteria are articles or reviews in English language. Therefore, 28 documents which are not articles or review and 9 non-English documents were excluded. Then 2 retracted and 1 duplicated publication were also cut out. To ensure that the retrieved articles are in line with our research topic, 703 documents uninvolved in breast cancer immunotherapy resistance were removed as well. Finally, 1341 publications totally, including 765 articles and 576 reviews were incorporated into the scientometric analysis. The plain text files of these publications were downloaded in the format “full record and cited references”.

### 2.2 Data analysis

The bibliometric analysis tools used in this study were Biblioshiny (Bibliometrix web interface) based on R version 4.3.1, VOSviewer software (version 1.6.18) and CiteSpace software (version 6.3.R1). Pajek software (version 5.18) was also used to adjust the clustering to make the images clearer.

Bibliometrix is an R package that offers statistical analysis techniques and a toolkit for network construction and visualization (32). VOSviewer, a Java-based software co-developed by Eck and Waltman, facilitates analysis of coupling, collaboration, co-occurrence, and co-citation analysis, and generates visual maps to help researchers quickly identify core literature and research hotspots within a specific field (33). Citespace software, also based on Java, was created by Dr. Chaomei Chen, a Chinese-American, utilizing citation analysis theory, which enables the visualization of the evolution and research frontiers within an academic field (34).

**Figure 1:**
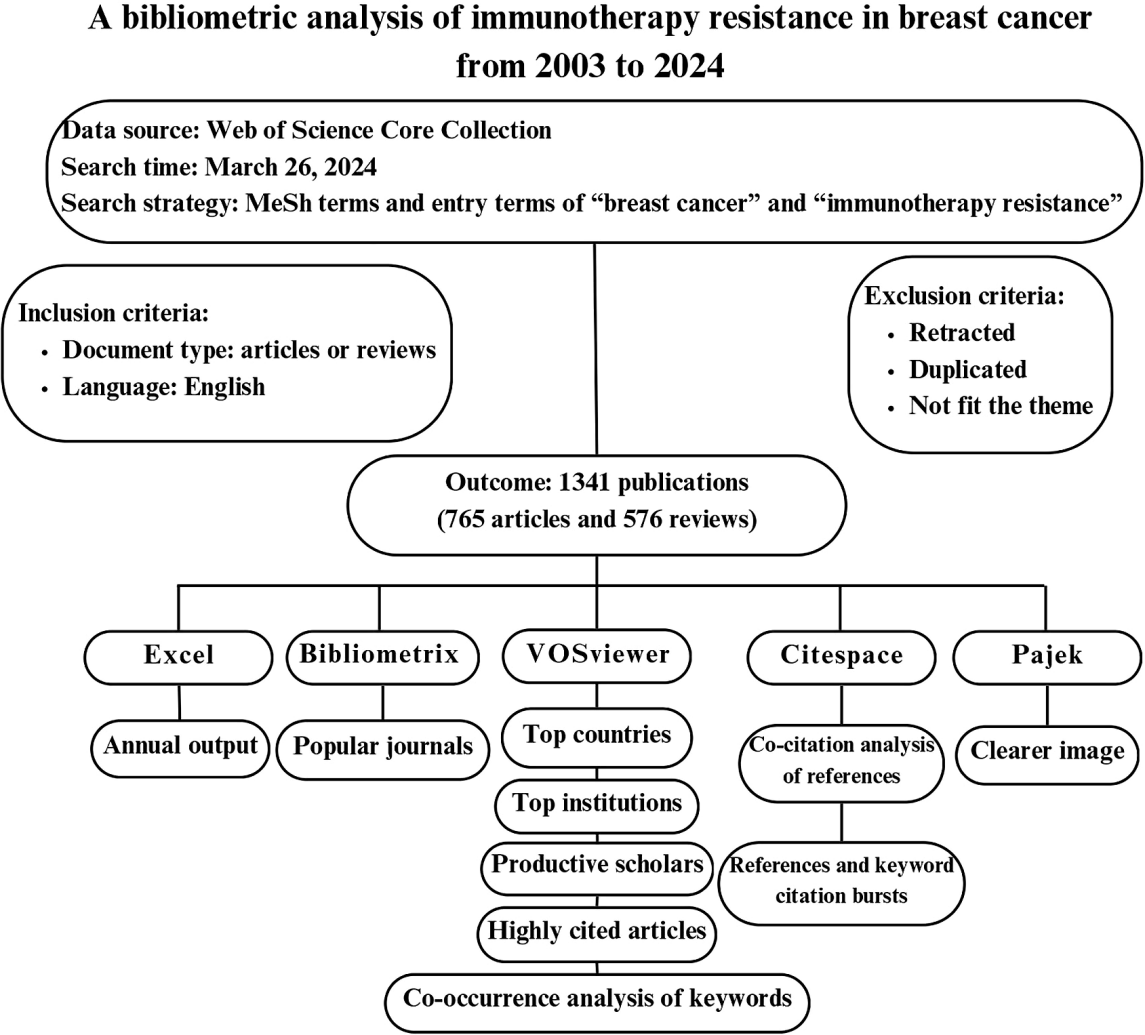
Flowchart of scientometric analysis process in breast cancer immunotherapy resistance study.

In this study, we initially generated a line chart using Excel 2024 to illustrate the publication outputs and citation trends of drug resistance in immunotherapy for breast cancer from 2003 to 2024. Using VOSviewer software, we identified the leading countries and institutions in the field and created a world map to visualize the distribution of productive countries. Bibliometrix was employed to map popular journals and their dynamic contributions. VOSviewer provided a list of the most productive and highly cited scholars. We assessed the quality of authors’ publications based on the number of publications and citations in the field and utilized journal impact factors from Journal Citation Report in 2019. Furthermore, we utilized CiteSpace to create a dual-map illustrating the relationship between cited journals and co-cited journals. VOSviewer and CiteSpace were employed for citation analysis of highly cited articles, co-citation analysis of references, and co-occurrence analysis of keywords. These analyses were presented in network diagrams and overlay maps. Additionally, Citespace software was employed to identify references and keyword citation bursts. Detailed explanations of the specific functions and operations can be found on the respective software’s websites, operation manuals or relevant literature. **Figure 1** presents a flowchart depicting the retrieval strategy, data collection and analysis process employed in this study.

## 3 Results

### 3.1 Publication and citation trends of breast cancer immunotherapy resistance

**Figure 2:**
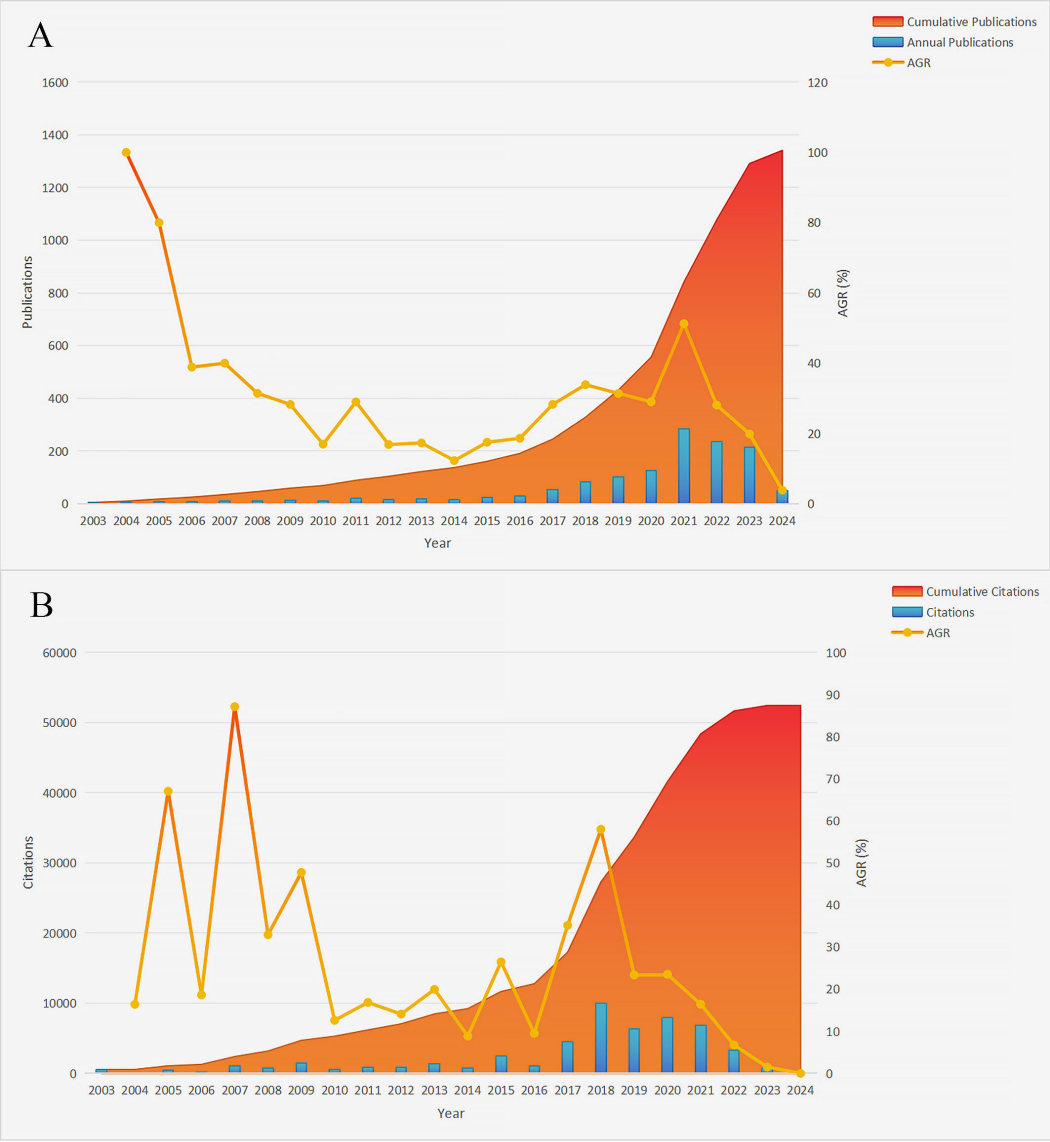
Annual publications, citations and respective cumulative values in the field of breast cancer immunotherapy resistance from 2003 to 2024. **(A)** Annual publications. **(B)** Annual citations (AGR, Annual growth rate).

**Figure 2** presents the annual trends in breast cancer immunotherapy resistance research. It includes a bar chart illustrating the number of publications (**Figure 2A**) or citations (**Figure 2B**), a line chart depicting the average growth rate, which is calculated using the formula: Average Growth Rate = [(Ending Value/Starting Value)^(1/Number of Periods)]–1 Equation 1 (35), and a stacked chart representing the cumulative value of publications or citations over time. In **Figure 2A**, a generally upward trend was observed from 2003 to 2016, with fluctuations in the annual growth rate. However, starting from 2017, the annual growth rate consistently remained high, reaching its peak in 2021 at 51.26%. Notably, 285 articles were published in 2021, indicating a substantial research output of that year. The past 7 years (2017–2023) have been pivotal for the development of this field, as evidenced by a sustained high level of annual publications, despite a slight decrease in the past three years. Noteworthy increases in publication numbers occurred in 2011, 2015, 2017 and 2021, suggesting the presence of potentially significant discoveries during these periods that warrant attention.

Average Growth Rate:


(1) Average Growth Rate=[(EndingValue/StartingValue)^(1/Number of Periods)]−1

As shown in **Figure 2B**, the number of annual citations in the field of breast cancer immunotherapy resistance experienced a sharp increase in 2018 but declined in 2023. The period spanning from 2018 to 2022 witnessed widespread citation of articles in this field, aligning with the upward trend observed in annual publication numbers. This further supports the notion that the past 7 years have been a golden period of development. Additionally, notable spikes in citation numbers occurred in 2005, 2007, 2009, 2015, 2017 and 2018, indicating significant research findings that garnered increased attention within the field.

### 3.2 Leading countries and organizations

**Figure 3:**
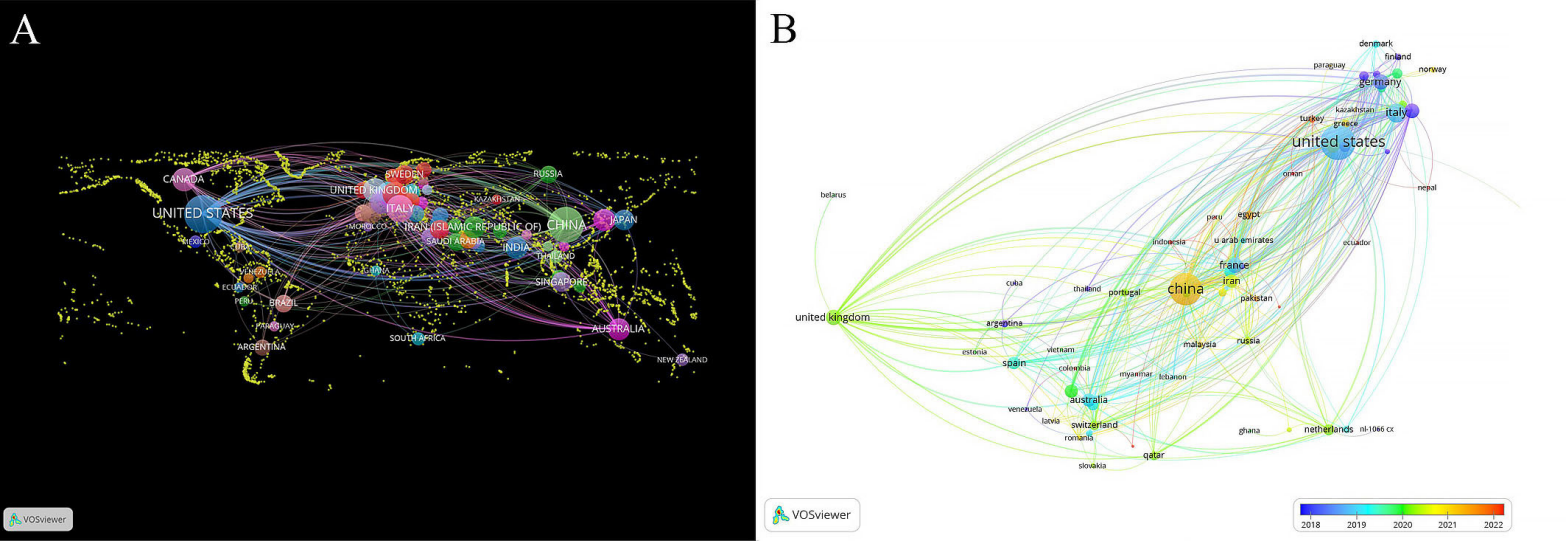
Distribution of countries and collaboration in immunotherapy resistance research for breast cancer treatment. **(A)** world map of productive countries’ distribution. **(B)** overlay map of countries’ average publication year.

In total, 70 countries actively participated in this field. The geographical distribution of these countries is visualized in **Figure 3**, where every circle symbolizes a unique country. The size of each circle corresponds to the quantity of documents published by that country, while the connections between circles indicate the strength of collaborations. As depicted in **Figure 3A**, the United States takes the lead with 517 articles, followed by China with 415 articles. Italy contributed 106 articles, while Germany, France, the United Kingdom, Canada, Australia, India and South Korea contributed 65, 62, 60, 54, 42, 41 and 40 documents respectively. **Figure 3B** highlights the recent contributions of countries with nodes closer to red indicating a more recent average publication year and nodes closer to blue suggesting relatively older work. The countries with the highest productivity levels have consistently shown activity in this field over the past five years. Additionally, China, Egypt, Greece and Turkey have made significant contributions in the last three years, as reflected by the increasing intensity of red shading. Regarding international collaboration, the United States stands out with the strongest ties to other countries, as indicated by a total link strength of 328. However, China (total link strength=143), the United Kingdom (total link strength=93), France (total link strength=92) and Italy (total link strength=92) lag behind. This highlights the importance of enhancing and fostering international collaboration among countries.

**Table 1** lists the top 11 institutions with highest number of publications output from 2003 to 2024. Harvard Medical School emerges as the leading institution with the most publications (47 papers) and citations (n=5668) and the strongest collaboration with other institutions (total link strength=110). University of Texas MD Anderson Cancer Center follows with 34 publications, indicating potential for further collaboration (total link strength=44). Dana-Farber Cancer Institute ranks third with 29 articles but excels in collaboration with other institutions (total link strength=77) and receives the second-highest number of citations (n=4867), which highlights its significant contributions towards advancing the development of this area. Among the top 11 institutions, five are from the United States, four from China, while the remaining two are from Italy and Singapore. This underscores the substantial research efforts from both the United States and China. However, it also emphasizes the need for China to strengthen collaboration with other countries to foster a broader international research network.

**Table 1:** Top 11 institutions with the highest publication outputs investigating resistance to immunotherapy in breast cancer from 2003 to 2024.

Rank	Institution	Publications	Citations	Total link strength
1	Harvard Medical School	47	5668	209
2	University of Texas MD Anderson Cancer Center	34	1798	91
3	Dana-Farber Cancer Institute	29	4867	140
4	Zhejiang University	26	1289	44
5	Sun Yat Sen University	24	1168	56
6	Sichuan University	23	479	13
7	Chinese Academy of Sciences	22	1248	65
8	University of Milan	21	577	83
9	Brigham & Women’s Hospital	20	2386	97
10	National University of Singapore	20	1300	102
11	National Cancer Institute (NCI)	20	614	77

### 3.3 Prominent Journals and authors

**Figure 4:**
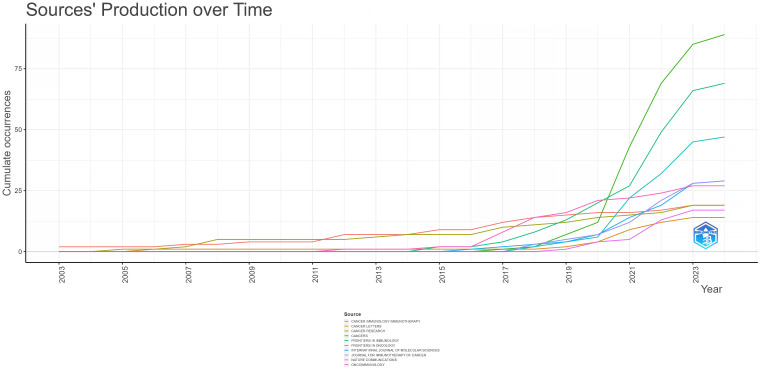
Source production on breast cancer immunotherapeutic resistance over time.

**Figure 5:**
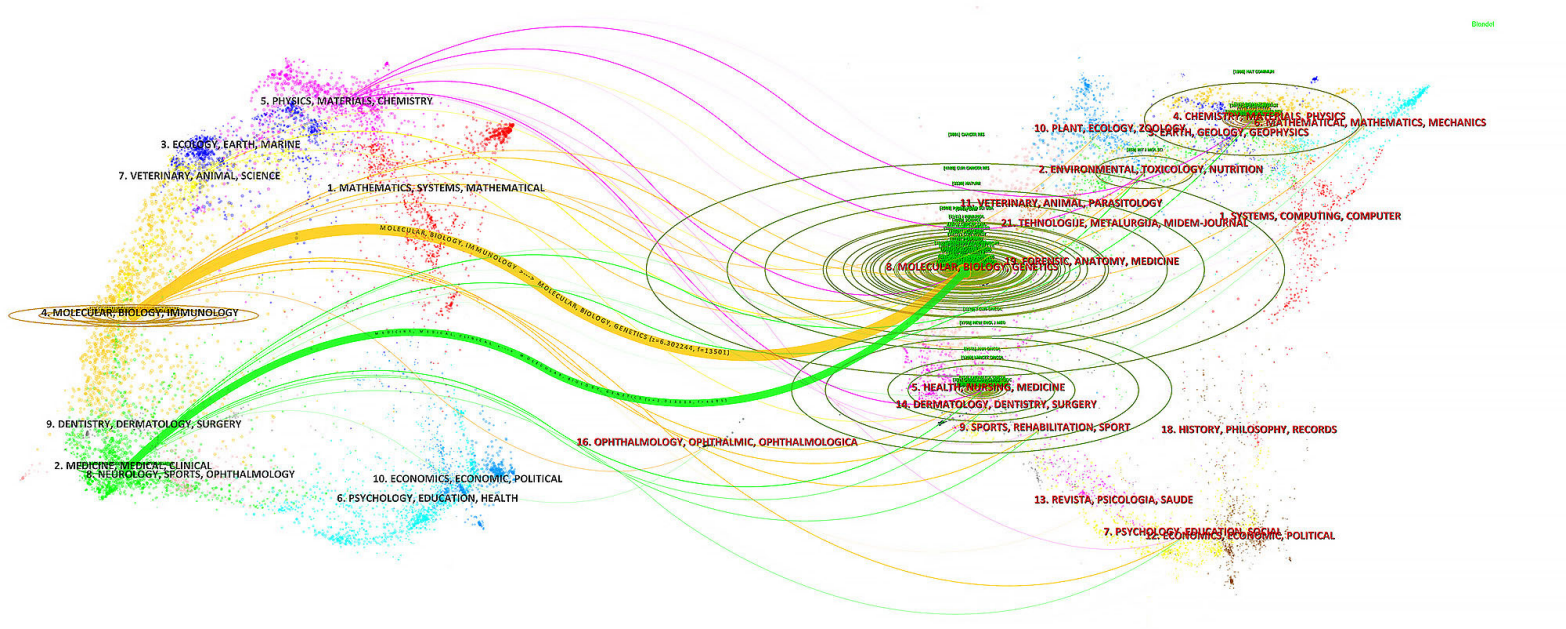
Dual-map of journals from citing documents to cited references.

In the ranking of journal publications output (**Table 2**), *Cancers* stands out by publishing 89 papers. Following closely are *Frontiers in Immunology* and *Frontiers in Oncology*, which have published 69 and 47 papers respectively. Tied at the fourth position, both *International Journal of Molecular Sciences* and *Journal for Immunotherapy of Cancer* have contributed significantly with 29 papers each. **Figure 4** demonstrates that *Cancer Immunology Immunotherapy*, *Cancer Research* and *Cancer Letters* have consistently made notable contributions to the field throughout the entire period from 2003 to 2024. Interestingly, the top 5 journals in terms of publications output began to gain prominence around 5-8 years ago and have quickly surpassed their counterparts. Based on citation count, *Nature Medicine* leads with 2430 citations, closely followed by *Nature Reviews Cancer*, which has accumulated 2201 citations. In the next positions, *Cancer Discovery* (n=1542), *Frontiers in Immunology* (n=1469) and *Clinical Cancer Research* (n=1432) have also received significant citations in their publications. **Figure 5** illustrates a dual-map of journals from citing documents to cited references. The citing journals are primarily from the fields of medicine, clinical research, and immunology, while the cited journals are predominantly from the fields of molecules, biology, and genetics.

**Table 2:** Top 10 journals with the most publications output related to breast cancer immunotherapy resistance in 2003-2024.

Rank	Journal	Publications	Citations	JCR	IF
1	Cancers	89	1351		
2	Frontiers in Immunology	69	1469		
3	Frontiers in Oncology	47	848		
4	International Journal of Molecular Sciences	29	383		
5	Journal for Immunotherapy of Cancer	29	652		
6	Oncoimmunology	27	875		
7	Cancer Immunology Immunotherapy	19	576		
8	Cancer Research	19	1327		
9	Nature Communications	17	636		
10	Cancer Letters	14	618		

As shown in **Table 3**, Curigliano, G has contributed the most significantly to the field, publishing a total of 12 relevant articles, followed by Tolaney, S. M. (n=11), Cavallo, F (n=8), Chouaib, S (n=8), Conti, L (n=8), De Lorenzo, C (n=8) and Mittendorf, E (n=8). Barroso-Sousa, R, Janji, B, Wang, J and Zhang, Y each published 7 articles, sharing the fourth position. From **Table 4**, Wucherpfennig, K. W. has garnered the highest citations in the field (total citations=2586), followed by Liu, J (total citations=2580), Jiang, P (total citations=2557), Liu, X. S. (total citations=2536) and Li, B (total citations=2441), despite their lower publication count (2-4 papers). Mittendorf, E. A. and Tolaney, S. M. rank highest in total link strength, indicating their higher level of collaboration with other authors. However, overall, the level of collaboration among researchers appears limited, suggesting a lack of effective cooperation within the academic community.

**Table 3:** Most prolific authors contributing to immunotherapy resistance research for breast cancer.

Authors	Number of publications	Number of citations	Total link strength
Curigliano, G	12	323	62
Tolaney, S. M.	11	353	120
Cavallo, F	8	232	70
Chouaib, S	8	1160	78
Conti, L	8	232	70
De Lorenzo, C	8	151	51
Mittendorf, E. A.	8	506	132
Barroso-Sousa, R	7	239	79
Janji, B	7	892	72
Wang, J	7	176	79
Zhang, Y	7	210	56

**Table 4:** Most cited authors contributing to immunotherapy resistance research for breast cancer.

Authors	Number of citations	Number of publications	Total link strength
Wucherpfennig, K. W.	2586	3	55
Liu, J	2580	2	42
Jiang, P	2557	4	52
Liu, X. S.	2536	3	36
Li, B	2441	3	21
Freeman, G. J.	2423	4	62
Bu, X	2409	2	14
Brown, M. A.	2409	1	25
Fu, JX	2409	1	14
Gu, SQ	2409	1	14
Hu, XH	2409	1	14
Li, ZY	2409	1	14
Pan, D	2409	1	14
Sahu, A	2409	1	14
Traugh, N	2409	1	14

### 3.4 Extensively cited literature and co-cited references

Top 10 most cited publications shed light on various aspects of this field (**Table 5**). Ranking first is an article published by Jiang et al. in 2018 in *Nature Medicine* (the most cited journal), which introduced the Tumor Immune Dysfunction and Exclusion model to effectively simulate the mechanisms of tumour immune evasion. The research predicted the response of multiple cancer types, including breast cancer to immune checkpoint blockade (ICB) therapy, and highlighted the significance of a novel ICB resistance modulator. The second most cited publication is a review by Hegde et al. published in *Immunity*, which discussed the top 10 challenges in cancer immunotherapy, emphasizing the complexity and modulation of the immune system in the context of cancer treatment. The article by Lee et al., ranking the third, explored the impact of radiotherapy on the immune microenvironment of breast cancer and put forward the notion of combining radiotherapy and ICB as a potential strategy to overcome immune resistance in breast cancer cells. In 2018, Tokunaga et al. discovered the role of the CXCL9, CXCL10, CXCL11/CXCR3 axis in immune cell migration, differentiation and activation. Their study suggests that the levels of CXCL9 and CXCL10 expression in breast cancer tissues is correlated with prognosis and tumor-infiltrating lymphocytes, which may influence immunotherapy response and also discussed the crosstalk between this axis and other immune pathways, such as the PD-1/PD-L1 axis. Another review, ranking the fifth in citations, summarizes various resistance mechanisms associated with breast cancer treatment, some of which are linked to immunotherapy resistance. The review highlights strategies to overcome resistance, such as using non-cross-resistant combination drugs, altering drug delivery methods and developing targeted therapies against signalling and apoptosis pathways. It emphasizes the importance of continued research on resistance mechanisms in breast cancer immunotherapy, with the integration of genomics and proteomics technologies.

**Table 5:** Top 10 most global cited documents in the field of breast cancer immunotherapy resistance from 2003 to 2024.

Rank	Documents	Year	First author	Journal	DOI	Total citations
1	Signatures of T cell dysfunction and exclusion predict cancer immunotherapy response	2018	Jiang P	Nature Medicine	10.1038/s41591-018-0136-1	2409
2	Top 10 Challenges in Cancer Immunotherapy	2020	Hegde PS	Immunity	10.1016/j.immuni.2019.12.011	1018
3	Therapeutic effects of ablative radiation on local tumor require CD8^+^ T cells: changing strategies for cancer treatment	2009	Lee YJ	Blood	10.1182/blood-2009-02-206870	1003
4	CXCL9, CXCL10, CXCL11/CXCR3 axis for immune activation - A target for novel cancer therapy	2018	Tokunaga R	Cancer Treatment Reviews	10.1016/j.ctrv.2017.11.007	743
5	Overview of resistance to systemic therapy in patients with breast cancer	2007	Gonzalez-Angulo AM	Advances in Experimental Medicine and Biology	10.1007/978-0-387-74039-3_1	703
6	Sphingolipid metabolism in cancer signalling and therapy	2018	Ogretmen B	Nature Reviews Cancer	10.1038/nrc.2017.96	671
7	Tumorigenic and Immunosuppressive Effects of Endoplasmic Reticulum Stress in Cancer	2017	Cubillos-Ruiz JR	Cell	10.1016/j.cell.2016.12.004	547
8	Microenvironmental regulation of therapeutic response in cancer	2015	Klemm F	Trends in Cell Biology	10.1016/j.tcb.2014.11.006	509
9	Combined antiangiogenic and anti-PD-L1 therapy stimulates tumor immunity through HEV formation	2017	Allen E	Science Translational Medicine	10.1126/scitranslmed.aak9679	505
10	Breast Cancer Immunotherapy: Facts and Hopes	2018	Emens LA	Clinical Cancer Research	10.1158/1078-0432.CCR-16-3001	495

**Figure 6:**
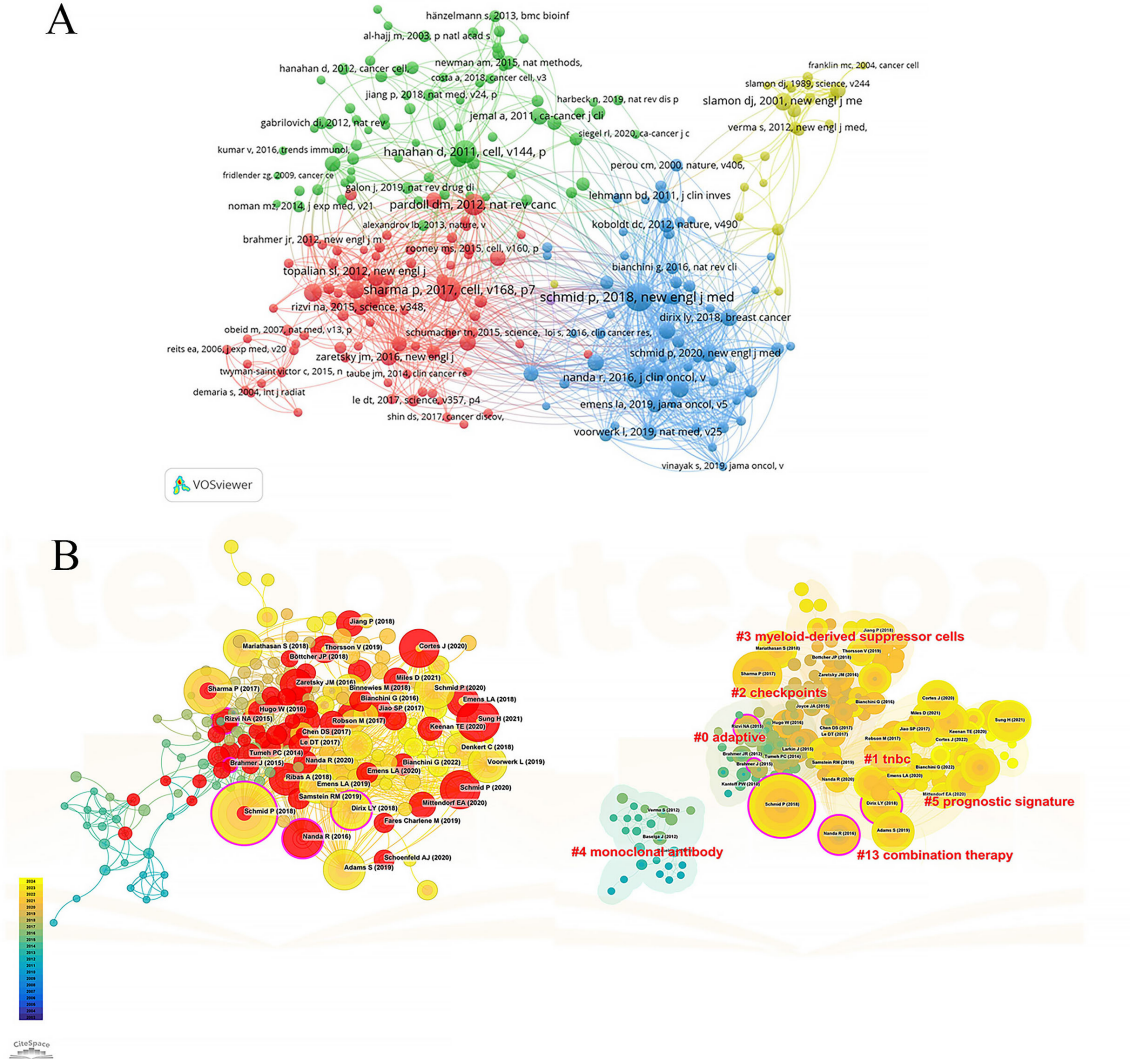
Co-citation analysis of cited references. **(A)** Network map of co-cited references. **(B)** Trend, centrality, citation burst and keyword clustering analysis of co-cited references.

Among the co-cited references in **Figure 6**, the highest in terms of citation count is the article by Schmid et al., published in the *New England Journal of Medicine* in 2018. This article has been cited for a total of 160 times, indicating the ability of the combination of atezolizumab and nab-paclitaxel to prolong progression-free survival in patients with advanced triple-negative breast cancer, particularly in the PD-L1 positive subgroup. **Figure 6A** is a network graph created by VOSviewer, where different colors represent different clusters of co-cited references, circle size represents the number of citations and the number of lines and distance between two circles represent the degree of connection. They can be roughly divided into four clusters, of which the red cluster is closely related to the blue and green clusters. **Figure 6B** drawn by CiteSpace software divides the references into seven clusters according to keywords, including: #0 adaptive, #1 tnbc, #2 checkpoints, #3 myeloid-derived suppressor cells, #4 monoclonal-antibody, #5 prognostic signature, #13 combination therapy. The lower the serial number, the greater the number of citations encompassed within that cluster. The nodes marked in red represents strong citation bursts and the larger the purple outer of the nodes, the stronger its intermediary centrality. The most cited reference also has the highest intermediary centrality (0.16), suggesting its significant bridging role in this field. Ranking second is the review by Sharma et al., published in *Cell* in 2017 with 126 citations, exploring the potential factors contributing to immunotherapy resistance, such as the tumor immune microenvironment, tumor antigen expression and adaptive resistance of tumors to immune attacks. It also proposes strategies to improve the effectiveness of immunotherapy by targeting tumor-associated macrophages (TAMs) and modulating tumor microenvironment. The article by Hanahan et al., ranks third with 114 citations. In 2012, the article by Pardoll et al. followed closely in fourth place with 103 citations. Ranking fifth is the study by Nanda et al., published in *Journal of Clinical Oncology* with 82 citations. Tumeh et al.’s article published in *Nature* in 2014, achieved the highest burst strength (14.9), maintaining its heat from 2015 to 2018. The article by Rizvi et al. published in 2015, exhibited the longest duration of burst with a burst strength of 11.61, extending from 2015 to 2020. It also holds an intermediary centrality of 0.15, indicating a significant bridging role and widespread citation in this field.

### 3.5 Topic evolution of popular keywords

**Figure 7:**
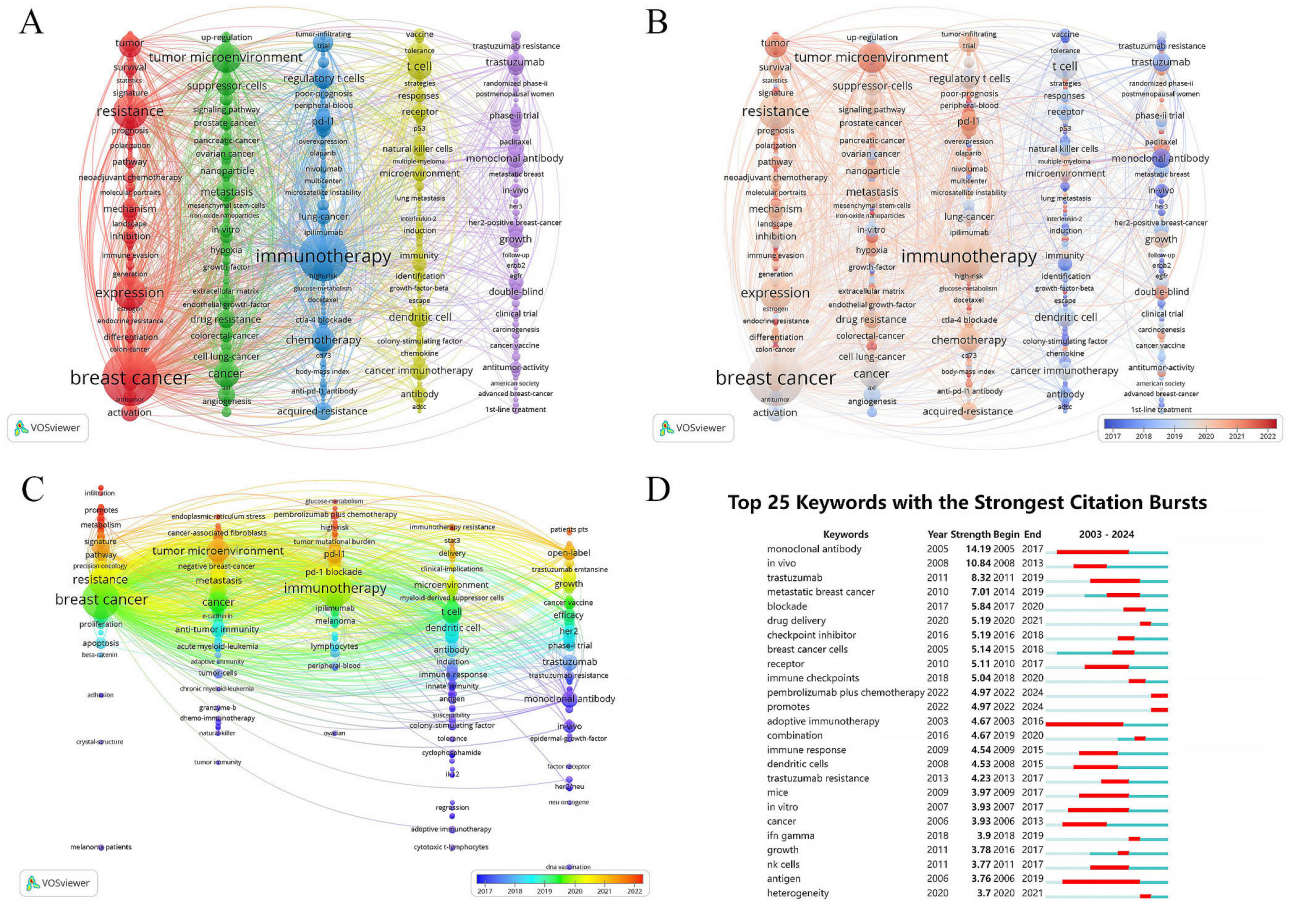
Cluster analysis and topic evolution of hot topics. **(A)** Network map of keywords occurring more than 5 times. **(B, C)** Overlay map of keywords based on average publication year. **(D)** Top 25 keywords with the strongest citation bursts.

A total of 493 out of 5,330 keywords appeared at least five times, forming the network graph of **Figure 7A**. The node size represents the frequency of keywords’ occurrence, while the distance between nodes indicates the strength of keywords’ connections. Top 10 keywords, ranked by frequency of occurrence, are “breast cancer”, “immunotherapy”, “resistance”, “expression”, “tumor microenvironment”, “cancer”, “T cell”, “therapy”, “chemotherapy” and “cell”. They are grouped into five clusters as shown below:

Cluster 1 (in red) includes “breast cancer”, “resistance”, “expression”, “cell”, “triple negative breast cancer” and so on. This cluster focuses on exploring the mechanisms of immune evasion in breast cancer through bioinformatics methods and investigating the interplay between immunotherapy and other treatment modalities such as endocrine therapy and chemotherapy. It also involves studying strategies for precise and personalized cancer management.

Cluster 2 (in green) comprises the keywords such as “tumor microenvironment”, “cancer”, “metastasis”, “suppressor-cells” and “drug resistance”. They investigate various signaling molecules and cells related to immune therapy resistance in breast cancer, such as cancer-associated fibroblasts, tumor stem cells, extracellular matrix and epithelial-mesenchymal transition. They also explore the role of combining techniques like photodynamic therapy, anti-angiogenic therapy, radiotherapy, and nanotechnology in enhancing immune efficacy.

Cluster 3 (in blue) includes “immunotherapy”, “chemotherapy”, “regulatory T cells”, “immune checkpoint inhibitor”, “tumor infiltrating lymphocytes” and so on. This cluster primarily focuses on evaluating the impact of immune checkpoint inhibitors on breast cancer prognosis through clinical research design.

Cluster 4 (in yellow) including “T cell”, “therapy”, “dendritic cell”, “cancer immunotherapy”, “receptor” and so on, investigates the role of immune cell-mediated cytotoxic responses in anti-tumor immune reactions through animal experiments.

Cluster 5 (in purple) includes the keywords “trastuzumab”, “growth”, “open-label”, “HER2” and “targeted therapy”, exploring clinical trials related to HER2-positive breast cancer treatment, such as antibody-drug conjugates, tyrosine kinase inhibitors and HER2-targeted monoclonal antibodies. It also involves cancer vaccines, CAR-T cell therapy and other approaches.

As shown in **Figure 7B**, Clusters 1-3 represent the most prominent areas of interest in the last five years. Representative keywords in these clusters include “roles”, “immune landscape”, “infiltration”, “glucose-metabolism” and “single-cell”, showing a focus on deeper understanding the biological mechanisms underlying immune therapy resistance in breast cancer through bioinformatics techniques, developing new biomarkers and treatment approaches to predict and manage patient prognosis. **Figure 7C** further illustrates the time trends of keywords within each cluster. Recent research hotspots include “infiltration”, “promotes” and “metabolism” in Cluster 1, “endoplasmic-reticulum stress”, “cancer-associated fibroblasts” and “tumor microenvironment” in Cluster 2, “glucose-metabolism”, “pembrolizumab plus chemotherapy” and “high-risk” in Cluster 3, “immunotherapy resistance”, “stat3” and “delivery” in Cluster 4, and “patients pts” and “open-label” in Cluster 5. These findings highlight the focus on molecular biological mechanisms related to immune therapy resistance, such as endoplasmic reticulum stress, cancer-associated fibroblasts and tumor microenvironment, as well as the impact of combined therapies and drug delivery systems on breast cancer immune therapy efficacy in recent years.

Using CiteSpace for citation burst detection of keywords has indeed contributed to identifying suddenly and widely recognized keywords, known as burst keywords, which are crucial metrics of research trends in the field (**Figure 7D**). The 25 keywords exhibiting the most significant citation bursts were divided into three categories: immune cells, antigens, and antibodies involved in the immune microenvironment; *in vitro* and *in vivo* experimental studies on immune resistance mechanisms; immune-related treatment methods and drugs. The keyword “adoptive immunotherapy” gained the earliest and most sustained attention, with a burst period from 2003 to 2016. “Monoclonal antibody” achieved the highest burst strength of 14.19, with a burst period from 2005 to 2017. Recently highlighted burst keywords include “drug delivery”, “heterogeneity”, “pembrolizumab plus chemotherapy” and “promotes”, emphasizing the focus on tumour heterogeneity, immune therapy resistance, the impact of drug delivery systems and combination therapies on immune therapy efficacy.

## 4 Discussion

### 4.1 Current landscape of research on drug resistance in immunotherapy for breast cancer

While immunotherapy has garnered widespread recognition in the field of breast cancer treatment, demonstrating remarkable therapeutic effects and enhancements in survival rates, only a portion of patients exhibits a favorable response to this approach (36). There are cases where patients develop resistance to immunotherapy, posing a challenge in achieving optimal outcomes for all individuals. To further advance drug development and improve the effectiveness of clinical trials, it is crucial to understand the progression of immunotherapy resistance in breast cancer treatment. This study is the first to examine the literature available in this area from 2003 to 2024, displaying trends in yearly publications and citations, participation of countries, organizations and authors, high-impact journals, core publications, key references, and hot keywords to elucidate the evolution of this significant area.

There has been a notable increase in the last 20 years in the realm of breast cancer immunotherapy resistance, from 5 documents at the beginning to 1341 at the end. The past seven years have been a period of rapid development marked by numerous significant discoveries that account for 82% of the total publications. Regarding country distribution, the United States and China have made significant contributions, with Harvard Medical School in the United States being not only the most prolific institution but also the most collaborative. Notably, the United States maintains its forefront position, boasting the highest number of clinical trials and immunotherapy drugs in development, while China and India have rapidly ascended in recent times. Analyzing highly cited publications and research focus reveals that China is intently engaged in developing predictive indicators and models for breast cancer immunotherapy response, alongside enhancing T-cell targeting efficacy (37–39). The United States, on the other hand, is deeply invested in unraveling the mechanisms of immune resistance and exploring strategies to overcome it (40, 41). India’s focus lies in the tumor microenvironment, delving into immune escape mechanisms of immune and stromal cells, and investigating the potential of nanotechnology in targeting cancer stem cells (42–44). Israel displays a keen interest in HER2 monoclonal antibodies and possesses robust research and development capabilities in innovative immunotherapy technologies (45). Likewise, Europe conducted numerous clinical trials in the past decade, and demonstrates a high level of activity in the approval and utilization of immune checkpoint inhibitors (46–49). However, the interaction and cooperation among other countries, including China, is relatively limited, indicating that enhanced collaboration could improve the quality of research efforts. The journals publishing these studies are mainly focused on clinical, immunological, and oncological aspects, indicating the need for further research on molecular biology and other basic medical sciences to provide a more profound comprehension of the underlying molecular processes.

The top three most productive authors are Curigliano, G, Tolaney, S. M. and Cavallo, F, coming from the European Institute of Oncology in Italy, Dana-Farber Cancer Institute in the United States, and the University of Torino in Italy, respectively. Curigliano, G has shown a keen interest in the mechanisms of immune escape in HER2-positive breast cancer and triple-negative breast cancer (TNBC), potential targets for immunotherapy, identification of tumor-associated antigens, and the application of biomarkers in immunotherapy, with a particular emphasis on immune checkpoint inhibitors (ICIs) (50–53). Tumor Mutational Burden (TMB) is a key focus of Tolaney, S. M.’s research, as it is linked to the load of tumor neoantigens, T cell infiltration, and response to ICIs (54). Additionally, he has also focused on hormone receptor-positive (HR+) breast cancer, which typically shows lower tumor-invasive lymphocytes and less responsiveness to ICIs compared to TNBC (55). Cavallo, F paid closed attention to “cancer stem cells” (CSCs), which are known to be resistant to traditional treatments, but eliminated by immunotherapy (56, 57). Wucherpfennig, K. W. from Dana-Farber Cancer Institute located in the United States accumulated the most cited instances and contributed mainly to the past five years. His first article published in 2018 gained the most citations (58). He also conducted a transcriptomic analysis collaborating with Tolaney, S. M (59). However, there is still a noticeable lack of connection among authors, highlighting the need for strengthening future collaboration and communication among them.

### 4.2 Advancements and promising directions

References provide the research background and development context of the field, serving as the cornerstone of this area. Prominent papers and keywords offer a wealth of knowledge on cutting-edge areas. Over the past two decades, popular terms included “resistance”, “expression”, “tumor microenvironment”, “cancer”, “T cell”, “therapy”, “chemotherapy” and “cell”. Three striking areas that have emerged as focal points in this field are immune escape mechanisms, biomarkers for predicting immunotherapy efficacy, and strategies to enhance the effectiveness of immunotherapy. Present research endeavors to comprehend the mechanisms of immune evasion in breast cancer through bioinformatics, basic experiments, and clinical trials and develop strategies to improve immunotherapy efficacy, including combination therapies and drug delivery system improvement as well as identify novel biomarkers to predict patient response. Extensive focus has been directed towards tumor microenvironment (TME) and tumor heterogeneity in immune resistance mechanisms.

The conceptual advancements achieved in the past decade have introduced two emerging features to the original six hallmarks of cancer: the reprogramming of energy metabolism and the evasion of immune destruction (60). In addition to cancer cells, tumors possess another layer of complexity known as the “tumor microenvironment”. This realization has spurred extensive research into understanding the relationship between TME and immune resistance (61). Tumor immune microenvironment encompasses a diverse array of components, including extracellular matrix, signaling molecules, immune cells, tumor cells, blood vessels and lymphatic vessels (62). Investigating and comprehending the roles and interactions of these elements have emerged as recent research hotspots.

#### 4.2.1 Mechanisms of immunotherapy resistance in breast cancer

Immune cells in the tumor microenvironment can be classified into tumor-promoting immune cells and anti-tumor immune cells. Tumor-promoting immune cells contribute to primary and/or adaptive drug resistance by suppressing anti-tumor immune responses (63). As one of the key immune cells in TME of HER2-positive breast cancer and TNBC, tumor-infiltrating lymphocytes (TILs), primarily consisting of CD8+ cytotoxic T cells, CD4+ helper T cells, regulatory T cells (Tregs) and NK cells, etc., indicates a better prognosis (64–67).

Tregs have attracted significant attention for their immunosuppressive effects in TNBC, serving a dual role of protecting against autoimmune diseases and inducing an immunosuppressive phenotype in TME (68–71). Treg/Th17 cell axis may play a crucial role in breast cancer development by suppressing the polarization of Th1 cells and effector CD8+ T cells toward inhibitory T cells. Manipulating the TME through therapeutics targeting the Treg/Th17 cell axis, such as monoclonal antibodies against TGF-β and IL-2, tyrosine kinase inhibitors like dasatinib, immunosuppressants such as tacrolimus, oncomicroRNA-based therapeutics and immune checkpoint inhibitors, holds promise for breast cancer immunotherapy (72). Tregs undergo metabolic reprogramming and express various chemokine receptors, such as CCR2, CCR4, CCR5, CCR6 and CCR8, which contribute to their intratumoral migration (73, 74). Strategies for targeting Tregs include monoclonal antibodies, small molecule inhibitors, chemotherapy, and natural therapeutics like curcumin and resveratrol, which may improve the prognosis of TNBC (75, 76). Hypoxia, common in solid tumors, promotes immune suppression and protects tumor cells (77). Hypoxia-inducible factor 1α (HIF-1α) increases Treg abundance by inducing FOXP3 (78). Blocking adenosine receptor A2AR expressed on tumor cells can reduce Treg infiltration and facilitate CD8+ T cells’ anti-tumor ability through debilitating hypoxic HIF-1α signaling (79). Overexpression of lymphocyte activating gene 3 (LAG-3) in Tregs, produce immunosuppressive cytokines and are linked to tumor progression and unfavorable outcomes (80). Comprehensive understanding of Tregs in the tumor microenvironment and the functional pathways of Tregs is crucial for their use as diagnostic and prognostic markers in TNBC. Future research application will focus on targeting Tregs-mediated immunosuppression. Another hot keyword, “Dendritic cells (DCs)” are specialized antigen-presenting cells, crucial for activating CD8+ T cells and promoting immune response in tumors (81). However, factors released by cancer cells that suppress the immune response, such as TGF-β, VEGF and IL-10, inhibit the maturation of DCs (82). Strategies like radiotherapy, endocrine therapy, combination therapies like chemotherapy with PD-1/PD-L1 inhibitors and DC-based vaccines aim to activate and mature DCs, enhancing immune response. Efforts have been made to restore the crosstalk between DCs and CD4+ T cells by promoting DC maturation, which has shown promising results in conferring potent immunity in breast cancer with CDK4/6 inhibitors (CDK4/6i) and immune checkpoint blockade (ICB) therapy (83, 84). The combination vaccine of HER2/neu-loaded bone marrow-derived dendritic cells (BM-DC) plus QS-21 and anti-PD-L1 monoclonal antibody has demonstrated synergistic antitumor activity and immune response against HER2-positive breast cancer in mice (85). Additionally, oncolytic nanohybrids combined with non-transgenic virus and immune checkpoint inhibitors have shown the ability to effectively stimulate DCs and macrophages in both *in vitro* and *in vivo* settings (86). These approaches hold potential as next-generation personalized anti-tumor immunotherapies, offering alternatives to inhibit tumor metastasis and recurrence. Future research will focus on personalized vaccines, improving DC targeting, enhancing T cell affinity and identifying prognostic biomarkers. Combining DC vaccines with other treatments holds promise as an effective strategy.

In addition to immune cells, cancer-associated fibroblasts (CAFs) are a significant component of TME and have gained attention in recent years. CAFs actively promote cancer invasion and treatment resistance by regulating processes such as angiogenesis, chronic inflammation, extracellular matrix remodeling (87). They control immune cells’ function in TME through cytokine/chemokine secretion and direct cell-cell interactions, as well as metabolism effects like supporting malignant cell growth through alanine secretion (88). CAFs can activate signaling pathways like Wnt/β-catenin and Notch, which contribute to maintaining breast cancer cell stemness. In turn, cancer stem cells (CSCs) modulate CAF activity through the Hedgehog signaling pathway (89). Heterogeneous subsets of CAFs, such as the CAF-S1 subset, foster a context that inhibits immune responses by drawing in and maintaining CD4+/CD25+ T lymphocytes and enhancing regulatory T cell differentiation while inhibiting T effector cell proliferation (90). Targeting CAFs through inhibitors of the Wnt/β-catenin, Notch or Hedgehog signaling pathways may offer therapeutic opportunities for breast cancer. However, it’s important to note that anti-tumor therapies can activate fibroblasts, leading to altered phenotypes and treatment resistance (91). Therefore, strategies that combine traditional anti-tumor drugs with agents targeting CAFs need to be developed. The diversity of CAFs across various breast cancer subtypes necessitates the selection of specific CAF-targeted therapies based on subtype-specific marker expression patterns (92). Future research should develop more precise targeted therapy strategies based on addressing the heterogeneity of CAFs.

Otherwise, recent studies have focused on epithelial-mesenchymal transition (EMT), vascular structures, chemokines and signaling molecules, endoplasmic reticulum (ER) stress, tumor surface antigens and intratumoral microbiomes in breast cancer immunotherapy resistance. Understanding these factors is crucial for deeper insights into immunotherapy resistance.

Epithelial-to-Mesenchymal Transition (EMT) is a crucial process in embryonic development, and its occurrence in epithelial carcinomas can lead to increased stemness, treatment resistance, and evasion of immune surveillance (93). Reversing EMT to overcome immune resistance holds promise as a therapeutic strategy. The migration of lymphocytes to tumor sites is essential for immune surveillance, and therapies that enhance lymphocyte adhesion and infiltration, such as combinations of anti-VEGFR2 and anti-PD-L1 antibodies, which activate lymphotoxin-beta receptor (LTbR) signaling and lead to the formation of high endothelial venules (HEVs), are being studied for their ability to boost the tumor immune response (94, 95). The recruitment of lymphocytes is directed by specific adhesion molecules and chemokines, with the CXCL9, -10, and -11/CXCR3 axis being particularly important, and is a determinant of anti-PD-1 therapy efficacy (94, 96). The presence of neoantigens due to genetic mutations can influence the immunogenicity of tumor cells, but a lack of these antigens can lead to resistance to ICIs (97). The MAL2 protein may also play a role in immune evasion by reducing antigen presentation on tumor cells (98). Increasing activation of “Signal transducer and activator of transcription 3 (STAT3)”, another key molecule in immune modulation, is linked to immunosuppression and drug resistance. Certain platinum (IV) complexes have shown potential in inhibiting the JAK2-STAT3 pathway, suggesting their value in overcoming resistance to immunotherapy (99). In addition, immune checkpoints like CTLA-4 (cytotoxic T-lymphocyte-associated protein 4) and PD-1 (programmed cell death protein 1) are critical for immune response regulation, and their upregulation on tumor cells can lead to adaptive immune resistance. The expression of PD-L1 has emerged as a significant biomarker for predicting responses to immunotherapy, as seen in clinical trials with PD-1 inhibitors (24, 100–103). The regression of tumors after therapeutic PD-1 blockade requires pre-existing CD8+ T cells, which are negatively regulated by PD-1/PD-L1-mediated adaptive immune resistance, suggesting that the level of tumor infiltration by CD8+ T cells may serve as a predictive indicator for the response to immunotherapy (102). In addition to the PD-1/PD-L1 pathway, there are several other immune checkpoint receptors and ligands, such as B7-H3 and B7-H4, whose upregulation on tumor cells or tumor-infiltrating cells may influence breast cancer immune escape (104). The dual role of endoplasmic reticulum (ER) stress in TME has been the subject of extensive research. Disruption of ER homeostasis results in the accumulation of misfolded proteins, triggering ER stress (105). While the endoplasmic reticulum stress response can inhibit the anti-cancer immune response by affecting the function of bone marrow cells in TME, causing tumor cells to release soluble factors, it can also trigger immunogenic cell death and promote an anti-tumor immune response, which inspired the idea of using endoplasmic reticulum stress to enhance the efficacy of standard chemotherapy and evolving cancer immunotherapies (106). Immune-related intratumoral microbiomes, such as Acidobillus, Succinomonas, Clostridium aminura and Pseudobacterium, function on breast cancer prognosis, abundance of tumor-infiltrating immune cells, and immunotherapy efficacy, emerging as an area of interest (107). However, additional research is required to investigate the relationship between microbial microbiota and breast cancer immunotherapy.

Increasing evidence suggests that metabolic dysregulation in cancer cells and TME holds significant importance in cancer progression, recurrence and metastasis and treatment response (108). Glucose deficiency can hinder immune cell glycolysis, leading to reduced IFN-γ production and cytotoxic T lymphocyte (CTL) function (109). High lactate levels contribute to immune evasion and poor prognosis in breast cancer. TME lactic acidosis, caused by nutrient depletion during tumor progression, can alter the function of anti-tumor immune cells and become a major driver of immune evasion in TNBC (110). Fatty acid metabolism has also been implicated in immunotherapy resistance, with high fatty acid metabolic index associated with an immunosuppressive TME in breast cancer (111). Sphingolipids, a class of lipids involved in cell signaling and membrane structure, can modulate immune cell activation, proliferation, migration and survival. Alterations in sphingolipid metabolism can affect the immune response against cancer cells and consequently impact the effectiveness of cancer immunotherapy (112). Studies have explored the impact of pyrimidine metabolism on immune checkpoints, tumor-infiltrating immune cells and cytokine levels. Pyrimidine metabolism index (PMI) has been proposed to predict the immunotherapy response of breast cancer patients (113). The NAD metabolic pathway is also implicated in the immune microenvironment of breast cancer, and NAD+ supplementation has shown potential in enhancing the anti-tumor effect of T cell-based immunotherapy (99). Long non-coding RNAs (lncRNAs) play a role in metabolic reprogramming and immune microenvironment remodeling, contributing to breast cancer resistance to immunotherapy. LncRNAs such as GATA3-AS1 and TINCR have been implicated in tumor progression, immune evasion and resistance to PD-L1 inhibitors (114). However, further clinical studies are needed to establish the relationship between lncRNAs and immunotherapy response.

Exploring these factors and developing targeted therapeutic strategies hold promise for improving treatment efficacy and overcoming drug resistance. There is significant heterogeneity in the immune composition across breast cancer subtypes and patients. Immune subtyping based on immune cell abundance and phenotype has identified subtypes that respond differently to immunotherapy. Stromal heterogeneity and cancer stem cells also contribute to the immunosuppressive microenvironment and treatment resistance (115). Understanding tumor heterogeneity and leveraging it for personalized treatments will require comprehensive analysis and machine learning approaches.

#### 4.2.2 Biomarkers for predicting immunotherapy efficacy to breast cancer

Several potential biological targets have been identified to predict a patient’s response to breast cancer immunotherapy. These targets include tumor-infiltrating lymphocytes (TILs), programmed death ligand protein-1 (PD-L1) expression levels, tumor mutational burden (TMB), microsatellite instability (MSI), IFN γ signature, B cell infiltration, as well as liquid biopsy markers like circulating tumor cells (CTCs) and cell-free DNA (cfDNA) (116–122). Specific genetic alterations such as JAK mutations, beta-2 microglobulin (B2M) mutations, PTEN deletion, and activation of the Wnt-β-catenin signaling pathway have also been investigated (123–126). These biomarkers reflect the immunogenicity of the tumor and the activation status of T cells, providing insights into a breast cancer patient’s potential response to immunotherapy. For instance, tumors with high TMB tend to have more neoantigens that can be recognized by the immune system (127). Additionally, the immunophenotype of the tumor, such as being inflammatory, immune-rejected, or immune-desert, plays a critical role in predicting treatment response (97). Standardized evaluation criteria for these biomarkers are currently lacking, and further research is needed to establish their clinical value.

Future research directions involve the development and validation of immunogene signatures, exploration of liquid biopsy markers and the establishment of predictive models that incorporate multiple biomarkers to improve the precision of predicting immunotherapy response. The relationship between the gut microbiome and breast cancer may emerge as another novel area for developing effective biomarkers.

#### 4.2.3 Improve the efficacy of breast cancer immunotherapy

The immunosuppressive tumor microenvironment presents a formidable hurdle for breast cancer immunotherapy, yet ongoing research endeavors relentlessly to explore multifaceted strategies to breach this barrier. One promising avenue involves augmenting T cell infiltration. Another strategy revolves around enhancing chemokine expression, either via chemotherapy-induced chemokines or by manipulating adhesion molecules such as integrins and selectins. Depletion or inhibition of regulatory T cells (Tregs) aims to mitigate their immunosuppressive influence, while bolstering dendritic cell function and CD4+ T cell help optimizes T cell priming and infiltration (128). Additionally, scientists are investigating methods to enhance the function of CAR-T cells, such as co-expression of costimulatory molecules, combination with immune checkpoint inhibitors, and the use of small molecule drugs and biochemicals (129, 130).

Combination therapies have been widely studied in recent years and offer a holistic approach to overcoming multiple obstacles within the tumor microenvironment, involving immune checkpoint inhibitors with other treatments like chemotherapy, radiotherapy, and targeted therapy, tumor vaccines, MEK inhibitors, CDK4/6 inhibitors, PARP inhibitors, IDO inhibitors, anti-angiogenic therapy and epigenetic therapy, which may overcome tumor immune escape and control metastases by enhancing or inducing new anti-tumor immune responses. For instance, the combination of atezolizumab and nab-paclitaxel has demonstrated the ability to prolong progression-free survival in patients with metastatic triple-negative breast cancer. Trastuzumab is shown to activate MyD88-dependent Toll-like receptor (TLR) signaling, leading to the release of type I IFNs and priming of adaptive IFN-γ–producing CD8+ T cells. When exposed to IFN-γ, tumor cells additionally exhibit the expression of the immunosuppressive ligand PD-L1, enabling the blockage of the PD-L1/PD-1 interaction as a strategy to harness the immune-mediated responses elicited by trastuzumab. That may be the reason why the combination of trastuzumab and pembrolizumab may have a synergistic effect in HER2-positive and PD-L1-positive metastatic breast cancer patients (27). Triple therapy, involving radiotherapy alongside anti-CTLA-4 and anti-PD-L1 antibodies, has exhibited notable effectiveness in breast cancer treatment, in which radiotherapy enhances the impact of immune checkpoint inhibitors. Furthermore, the integration of photodynamic therapy (PDT) and photothermal therapy (PTT) with immunotherapy can augment both local and systemic immune responses, ultimately improving tumor control. An illustrative example is the utilization of gold nanoparticles for PTT, which, when combined with immune  checkpoint inhibitors, can amplify T-cell infiltration and activity (131–137). Anti-angiogenic drugs can improve immune penetration of the tumor microenvironment, thus enhancing the efficacy of immunotherapy (95). The PI3K-AKT-mTOR pathway, in addition to its direct effect on tumor cells, is involved in creating an immunosuppressive tumor microenvironment. Combining PI3K inhibitors with immunotherapy may enhance T cell-mediated tumor killing by increasing the CD8+/Treg ratios (138). Further understanding of the PI3K signaling pathway and its interactions with related pathways, as well as patient stratification and selection strategies, are important for the clinical development of PI3K inhibitors and their combination with immunotherapy.

Targeting specific immune escape mechanisms, such as the TGF β and Wnt/β-catenin signaling pathways, is another important approach (139). By intervening in altered metabolic pathways between immune cells and tumor cells, a more favorable environment for immune cells can be created (140). Synthetic immune methods like CAR-T cells and CD3 bispecific antibodies, which bind T cells to cancer cells, can trigger robust immune responses (141, 142). Considering the initiation and activation of immune cells, cytotoxic activity, the formation of memory responses, optimizing treatment sequencing and schedules is another avenue to explore. Targeting specific cell types such as CAFs and TAMs or cytokines such as IL-6 and TGF-β in TME can improve immune cell function (143). Personalized vaccines based on a patient’s unique tumor neoantigens can enhance the immune system’s recognition of the tumor (144). Complex biomarkers can be utilized to customize personalized treatment plans for each patient. Understanding the mechanisms of immunotoxicity and optimizing management, such as the use of corticosteroids and immunosuppressants, is crucial for balancing treatment efficacy and toxicity.

Estrogen, acting as an immunosuppressive factor, promotes tumor development in breast cancer. Anti-estrogen therapies not only directly kill cancer cells but also boost the immunogenicity of breast cancer cells and improve the infiltration and function of anti-tumor immune cells (145). Various endocrine therapy strategies, including SERD, SERM, AI, GnRHa, and inhibitors of PI3K, AKT, mTOR, and CDK4/6, are being explored to enhance the immune response in breast cancer (146). However, the potential therapeutic benefits of combining immunotherapy drugs with standard anti-estrogen therapy must be carefully weighed against the risk of toxicity. It is crucial to understand the immunosuppressive characteristics of luminal breast cancer and developing personalized treatment plans for different breast cancer subtypes.

Epigenetic modifications play a role in cancer progression and resistance to immunotherapy by altering cellular phenotypes and remodeling the tumor microenvironment (147). LSD1, a histone demethylase, is involved in various cellular processes in cancer and can enable tumor cells to overcome immune surveillance. Clinical trials of LSD1 inhibitors are underway, primarily in small cell lung cancer and acute myeloid leukemia, but their application in solid malignancies, including breast cancer, is limited (148–150). LSD1 may serve as a potential therapeutic target to overcome immunotherapy resistance, and further research is needed to understand its function in cancer epigenetics and its role in regulating tumor immunogenicity.

Drug delivery systems, particularly utilizing nanoparticles, plays a vital role in breast cancer immunotherapy (151). Surface-modified nanoparticles can enhance tumor cell targeting, increase local drug concentrations, reduce damage to normal cells, and minimize the development of drug resistance. The utilization of advanced nanoparticles, encompassing liposomes and polymeric nanoparticles, facilitates the precise and sustained liberation of antigens and adjuvants, thereby eliciting robust immune responses (152, 153). Furthermore, nanotechnology harnesses the potential to manipulate the immunosuppressive microenvironment within tumors, transforming ‘cold’ tumors into highly receptive ‘hot’ tumors that are more amenable to immunotherapy. Preclinical studies are yielding promising results from innovative strategies such as *in situ* gene delivery and the employment of extracellular matrix peptides for targeted antibody delivery (154). Nanocarriers can also activate immune cells, such as dendritic cells, to increase immunogenic cell death and enhance the immune system’s recognition and attack on tumors (155). Nanotechnology can regulate gene expression and enhance the synergistic effects of drugs and genes. Moreover, the integration of nanotechnology with traditional therapeutic modalities like chemotherapy, radiotherapy, and photodynamic therapy demonstrates a synergistic effect, resulting in intensified immune responses and superior treatment outcomes (151, 156, 157). Nanovaccines can be developed to stimulate specific immune responses against breast cancer by delivering tumor antigens and immune adjuvants (158). The realm of nano-immunoimaging is being actively explored, leveraging superparamagnetic iron oxide nanoparticles for the non-invasive and real-time monitoring of immune cells within living organisms, further advancing the precision and effectiveness of immunological interventions (154). Despite the tremendous potential of nanotechnology in breast cancer treatment, several research gaps persist, for example, assessing the long-term safety and biocompatibility of nanoparticles. Additionally, designing personalized nanotherapy protocols based on individual patient conditions and overcoming the challenge of translating laboratory findings into industrial production are current obstacles. Bridging these gaps is crucial for advancing nanotechnology in breast cancer treatment and necessitates interdisciplinary collaboration and innovative approaches.

While TIL therapy emerges as a promising frontier in breast cancer immunotherapy, harnessing the patient’s own T cells to specifically target and eliminate tumor cells, this innovative approach confronts several formidable obstacles, including intricate manufacturing protocols, substantial financial burdens, the imperative for individualization, the immunosuppressive nature of the tumor microenvironment, and uncertainties surrounding treatment outcomes’ predictability. Furthermore, meticulous monitoring of TIL therapy’s toxicity and side effects is crucial (65). Future research directions encompass the exploration of novel and distinctive targets, enhancing the migration and penetration of effector cells into tumor sites, improving the persistence and anti-tumor response of effector cells through cell engineering, and conducting additional clinical trials to validate laboratory research findings and assess the safety and efficacy of emerging therapies in actual patients.

### 4.3 Limitations

It is undeniable that there are certain flaws in this bibliometric analysis. Firstly, due to technical limitations and challenges in integrating results from different databases with varying formats, we were only able to download the necessary documents from Web of Science. Furthermore, our inclusion criteria focused solely on English-language documents, potentially omitting relevant studies from other languages and leading to incomplete coverage. However, we implemented a rigorous filtering process to ensure the inclusion of articles closely aligned with the research topic. Bibliometrix cannot accurately distinguish between authors with the same abbreviated name, which hinders our presenting the publication trend of individual authors over time. However, we attempted to compensate for this limitation by utilizing VOSviewer to provide a general overview of the period during which each author made significant contributions. Additionally, a small proportion of references and keywords may be missing, but this is unlikely to have a significant impact on the overall results. It is worth noting that the impact of recently published high-quality articles may be underestimated due to the continuous updating of the Web of Science Core Collection database, as these articles may not have accumulated enough citations at the time of analysis. As the evaluation of literature quality in bibliometric analysis is not as rigorous as in systematic reviews, there may be a potential misestimation of the impact of certain articles. Nevertheless, this paper aims to provide a broad overview of the research field and can serve as a guiding and generalizing resource.

## 5 Conclusions

The past two decades witnessed an escalating attention to immunotherapy resistance in breast cancer research, with a significant surge in the last seven years. The United States and China have emerged as major contributors, with Harvard Medical School leading the way as the most prolific institution. Influential authors such as Curigliano, G and Wucherpfennig, K. W. have made notable contributions. However, there is still a need for stronger connections and collaboration among countries and authors to enhance the quality of research. *Cancers* stands out as the most active journal in this area. Key topics of interest include understanding the mechanisms of immune escape in breast cancer through bioinformatics, basic experiments and clinical trials. Developing strategies to improve immunotherapy efficacy and identifying new biomarkers to predict patient response are also prominent areas of investigation. Overcoming resistance to immunotherapy in breast cancer and providing better treatment options for patients are crucial areas for future research and development.

## Data Availability

The original contributions presented in the study are included in the article/supplementary material. Further inquiries can be directed to the corresponding authors.

## Glossary

**Table:**

TNBC	triple-negative breast cancers
HER2	human epidermal growth factor receptor 2
IARC	International Agency for Research on Cancer
ER	estrogen receptor
PR	progesterone receptor
ORRs	objective response rates
TILs	tumor infiltrating lymphocytes
PFS	progression-free survival
OS	overall survival
SCI-EXPANDED	Science Citation Index Expanded
SSCI	Social Sciences Citation Index
AHCI	Arts & Humanities Citation Index
ESCI	Emerging Sources Citation Index
CCR-EXPANDED	Current Chemical Reactions
IC	Index Chemicus
Mesh	Medical subject headings
AGR	average growth rate
ICB	immune checkpoint blockade
TAMs	tumor-associated macrophages
CAR-T	Chimeric Antigen Receptor T-cell
ICIs	immune checkpoint inhibitors
TMB	tumor mutational burden
HR+	hormone receptor-positive
Tregs	regulatory T cells
MDSCs	myeloid-derived suppressor cells
NK	natural killer
DCs	dendritic cells
CSCs	cancer stem cells
TME	tumor microenvironment
HIF-1α	hypoxia-inducible factor 1α
BM-DC	bone marrow-derived dendritic cells
CAFs	cancer-associated fibroblasts
EMT	Epithelial-mesenchymal transition
ER	endoplasmic reticulum
VEGF	vascular endothelial growth factor
LTbR	lymphotoxin-beta receptor
HEV	high endothelial venules
STAT3	signal transducer and activator of transcription 3
CTLA-4	cytotoxic T-lymphocyte-associated protein 4
PD-1	programmed cell death protein 1
CTL	cytotoxic T lymphocyte
PMI	Pyrimidine metabolism index
lncRNAs	Long non-coding RNAs
MSI	microsatellite instability
CTCs	circulating tumor cells
cfDNA	cell-free DNA
B2M	beta-2 microglobulin
AKT	Protein Kinase B
mTOR	mammalian Target of Rapamycin
SERD	selective estrogen receptor degrader
SERM	selective estrogen receptor modulator
CDK4/6	Cyclin-dependent kinases 4 and 6
PI3K	Phosphatidylinositol-3-kinase
PDT	photodynamic therapy
PTT	photothermal therapy

## References

[1] Bray F Laversanne M Sung H Ferlay J Siegel RL Soerjomataram I. Global cancer statistics 2022: GLOBOCAN estimates of incidence and mortality worldwide for 36 cancers in 185 countries. CA Cancer J Clin. (2024) 74(3):229–63. doi: 10.3322/caac.21834 38572751

[2] Łukasiewicz S Czeczelewski M Forma A Baj J Sitarz R Stanisławek A. Breast cancer-epidemiology, risk factors, classification, prognostic markers, and current treatment strategies-an updated review. Cancers (Basel). (2021) 13:4287. doi: 10.3390/cancers13174287 34503097 PMC8428369

[3] Sabatier R Finetti P Guille A Adelaide J Chaffanet M Viens P. Claudin-low breast cancers: clinical, pathological, molecular and prognostic characterization. Mol Cancer. (2014) 13:228. doi: 10.1186/1476-4598-13-228 25277734 PMC4197217

[4] Herschkowitz JI Simin K Weigman VJ Mikaelian I Usary J Hu Z. Identification of conserved gene expression features between murine mammary carcinoma models and human breast tumors. Genome Biol. (2007) 8:R76. doi: 10.1186/gb-2007-8-5-r76 17493263 PMC1929138

[5] Perou CM Sørlie T Eisen MB van de Rijn M Jeffrey SS Rees CA. Molecular portraits of human breast tumours. Nature. (2000) 406:747–52. doi: 10.1038/35021093 10963602

[6] Blows FM Driver KE Schmidt MK Broeks A van Leeuwen FE Wesseling J. Subtyping of breast cancer by immunohistochemistry to investigate a relationship between subtype and short and long term survival: a collaborative analysis of data for 10,159 cases from 12 studies. PloS Med. (2010) 7:e1000279. doi: 10.1371/journal.pmed.1000279 20520800 PMC2876119

[7] Osborne CK Schiff R. Estrogen-receptor biology: continuing progress and therapeutic implications. J Clin Oncol. (2005) 23:1616–22. doi: 10.1200/JCO.2005.10.036 15755967

[8] von Minckwitz G Procter M de Azambuja E Zardavas D Benyunes M Viale G. Adjuvant pertuzumab and trastuzumab in early HER2-positive breast cancer. N Engl J Med. (2017) 377:122–31. doi: 10.1056/NEJMoa1703643 PMC5538020 28581356

[9] Arteaga CL Sliwkowski MX Osborne CK Perez EA Puglisi F Gianni L. Treatment of HER2-positive breast cancer: current status and future perspectives. Nat Rev Clin Oncol. (2011) 9:16–32. doi: 10.1038/nrclinonc.2011.177 22124364

[10] Burguin A Diorio C Durocher F. Breast cancer treatments: updates and new challenges. J Pers Med. (2021) 11:808. doi: 10.3390/jpm11080808 34442452 PMC8399130

[11] Pazaiti A Fentiman IS. Basal phenotype breast cancer: implications for treatment and prognosis. Womens Health (Lond). (2011) 7:181–202. doi: 10.2217/WHE.11.5 21410345

[12] Lehmann BD Bauer JA Chen X Sanders ME Chakravarthy AB Shyr Y. Identification of human triple-negative breast cancer subtypes and preclinical models for selection of targeted therapies. J Clin Invest. (2011) 121:2750–67. doi: 10.1172/JCI45014 PMC3127435 21633166

[13] Li X Yang J Peng L Sahin AA Huo L Ward KC. Triple-negative breast cancer has worse overall survival and cause-specific survival than non-triple-negative breast cancer. Breast Cancer Res Treat. (2017) 161:279–87. doi: 10.1007/s10549-016-4059-6 27888421

[14] Weil RJ Palmieri DC Bronder JL Stark AM Steeg PS. Breast cancer metastasis to the central nervous system. Am J Pathol. (2005) 167:913–20. doi: 10.1016/S0002-9440(10)61180-7 PMC1603675 16192626

[15] Brown DM Ruoslahti E. Metadherin, a cell surface protein in breast tumors that mediates lung metastasis. Cancer Cell. (2004) 5:365–74. doi: 10.1016/S1535-6108(04)00079-0 15093543

[16] Park M Kim D Ko S Kim A Mo K Yoon H. Breast cancer metastasis: mechanisms and therapeutic implications. Int J Mol Sci. (2022) 23:6806. doi: 10.3390/ijms23126806 35743249 PMC9224686

[17] Saphner T Tormey DC Gray R. Annual hazard rates of recurrence for breast cancer after primary therapy. J Clin Oncol. (1996) 14:2738–46. doi: 10.1200/JCO.1996.14.10.2738 8874335

[18] Valabrega G Montemurro F Aglietta M. Trastuzumab: mechanism of action, resistance and future perspectives in HER2-overexpressing breast cancer. Ann Oncol. (2007) 18:977–84. doi: 10.1093/annonc/mdl475 17229773

[19] Osborne CK Schiff R. Mechanisms of endocrine resistance in breast cancer. Annu Rev Med. (2011) 62:233–47. doi: 10.1146/annurev-med-070909-182917 PMC3656649 20887199

[20] Coley HM. Mechanisms and strategies to overcome chemotherapy resistance in metastatic breast cancer. Cancer Treat Rev. (2008) 34:378–90. doi: 10.1016/j.ctrv.2008.01.007 18367336

[21] Baxevanis CN Perez SA Papamichail M. Cancer immunotherapy. Crit Rev Clin Lab Sci. (2009) 46:167–89. doi: 10.1080/10408360902937809 19650714

[22] Székely B Silber AL Pusztai L. New therapeutic strategies for triple-negative breast cancer. Oncol (Williston Park). (2017) 31:130–7. 28205193

[23] Basile D Pelizzari G Vitale MG Lisanti C Cinausero M Iacono D. Atezolizumab for the treatment of breast cancer. Expert Opin Biol Ther. (2018) 18:595–603. doi: 10.1080/14712598.2018.1469619 29690797

[24] Nanda R Chow LQ Dees EC Berger R Gupta S Geva R. Pembrolizumab in patients with advanced triple-negative breast cancer: phase ib KEYNOTE-012 study. J Clin Oncol. (2016) 34:2460–7. doi: 10.1200/JCO.2015.64.8931 PMC6816000 27138582

[25] Kwapisz D. Pembrolizumab and atezolizumab in triple-negative breast cancer. Cancer Immunol Immunother. (2021) 70:607–17. doi: 10.1007/s00262-020-02736-z PMC10992894 33015734

[26] Poudel P Nyamundanda G Patil Y Cheang MCU Sadanandam A. Heterocellular gene signatures reveal luminal-A breast cancer heterogeneity and differential therapeutic responses. NPJ Breast Cancer. (2019) 5:21. doi: 10.1038/s41523-019-0116-8 31396557 PMC6677833

[27] Stagg J Loi S Divisekera U Ngiow SF Duret H Yagita H. Anti-ErbB-2 mAb therapy requires type I and II interferons and synergizes with anti-PD-1 or anti-CD137 mAb therapy. Proc Natl Acad Sci U S A. (2011) 108:7142–7. doi: 10.1073/pnas.1016569108 PMC3084100 21482773

[28] Krasniqi E Barchiesi G Pizzuti L Mazzotta M Venuti A Maugeri-Saccà M. Immunotherapy in HER2-positive breast cancer: state of the art and future perspectives. J Hematol Oncol. (2019) 12:111. doi: 10.1186/s13045-019-0798-2 31665051 PMC6820969

[29] Retecki K Seweryn M Graczyk-Jarzynka A Bajor M. The immune landscape of breast cancer: strategies for overcoming immunotherapy resistance. Cancers. (2021) 13:32. doi: 10.3390/cancers13236012 PMC8657247 34885122

[30] Labani-Motlagh A Ashja-Mahdavi M Loskog A. The tumor microenvironment: A milieu hindering and obstructing antitumor immune responses. Front Immunol. (2020) 11:940. doi: 10.3389/fimmu.2020.00940 32499786 PMC7243284

[31] Bornmann L Leydesdorff L. Scientometrics in a changing research landscape: bibliometrics has become an integral part of research quality evaluation and has been changing the practice of research. EMBO Rep. (2014) 15:1228–32. doi: 10.15252/embr.201439608 PMC4264924 25389037

[32] Aria M Alterisio A Scandurra A Pinelli C D’Aniello B. The scholar’s best friend: research trends in dog cognitive and behavioral studies. Anim Cogn. (2021) 24:541–53. doi: 10.1007/s10071-020-01448-2 PMC8128826 33219880

[33] van Eck NJ Waltman L. Software survey: VOSviewer, a computer program for bibliometric mapping. Scientometrics. (2010) 84:523–38. doi: 10.1007/s11192-009-0146-3 PMC2883932 20585380

[34] Synnestvedt MB Chen C Holmes JH. CiteSpace II: visualization and knowledge discovery in bibliographic databases. AMIA Annu Symp Proc. (2005) 2005:724–8. PMC1560567 16779135

[35] Guo Y Hao Z Zhao S Gong J Yang F. Artificial intelligence in health care: bibliometric analysis. J Med Internet Res. (2020) 22:e18228. doi: 10.2196/18228 32723713 PMC7424481

[36] Nahta R Yu D Hung MC Hortobagyi GN Esteva FJ. Mechanisms of disease: understanding resistance to HER2-targeted therapy in human breast cancer. Nat Clin Pract Oncol. (2006) 3:269–80. doi: 10.1038/ncponc0509 16683005

[37] Zheng XC Fang ZX Liu XM Deng SM Zhou P Wang XX. Increased vessel perfusion predicts the efficacy of immune checkpoint blockade. J Clin Invest. (2018) 128:2104–15. doi: 10.1172/JCI96582 PMC5957454 29664018

[38] Wei J Sun HY Zhang AM Wu XJ Li YX Liu JW. A novel AXL chimeric antigen receptor endows T cells with anti-tumor effects against triple negative breast cancers. Cell Immunol. (2018) 331:49–58. doi: 10.1016/j.cellimm.2018.05.004 29935762

[39] Xie Y Chen Y Ahmed KA Li W Ahmed S Sami A. Potent CD4+ T-cell epitope P30 enhances HER2/neu-engineered dendritic cell-induced immunity against Tg1-1 breast cancer in transgenic FVBneuN mice by enhanced CD4+ T-cell-stimulated CTL responses. Cancer Gene Ther. (2013) 20:590–8. doi: 10.1038/cgt.2013.60 24052129

[40] Dongre A Rashidian M Reinhardt F Bagnato A Keckesova Z Ploegh HL. Epithelial-to-mesenchymal transition contributes to immunosuppression in breast carcinomas. Cancer Res. (2017) 77:3982–9. doi: 10.1158/0008-5472.CAN-16-3292 PMC5541771 28428275

[41] Kim IS Gao Y Welte T Wang H Liu J Janghorban M. Immuno-subtyping of breast cancer reveals distinct myeloid cell profiles and immunotherapy resistance mechanisms. Nat Cell Biol. (2019) 21:1113. doi: 10.1038/s41556-019-0373-7 31451770 PMC6726554

[42] Mehraj U Ganai RA Macha MA Hamid A Zargar MA Bhat AA. The tumor microenvironment as driver of stemness and therapeutic resistance in breast cancer: New challenges and therapeutic opportunities. Cell Oncol. (2021) 44:1209–29. doi: 10.1007/s13402-021-00634-9 PMC12980687 34528143

[43] Biswas S Mandal G Chowdhury SR Purohit S Payne KK Anadon C. Exosomes produced by mesenchymal stem cells drive differentiation of myeloid cells into immunosuppressive M2-polarized macrophages in breast cancer. J Immunol. (2019) 203:3447–60. doi: 10.4049/jimmunol.1900692 PMC6994919 31704881

[44] Malla RR Kamal MA. ROS-responsive nanomedicine: towards targeting the breast tumor microenvironment. Curr Med Chem. (2021) 28:5674–98. doi: 10.2174/0929867328666201209100659 33297907

[45] Zsebik B Citri A Isola J Yarden Y Szöllosi J Vereb G. Hsp90 inhibitor 17-AAG reduces ErbB2 levels and inhibits proliferation of the trastuzumab resistant breast tumor cell line JIMT-1. Immunol Lett. (2006) 104:146–55. doi: 10.1016/j.imlet.2005.11.018 16384610

[46] Buisseret L Pommey S Allard B Garaud S Bergeron M Cousineau I. Clinical significance of CD73 in triple-negative breast cancer: multiplex analysis of a phase III clinical trial. Ann Oncol. (2018) 29:1056–62. doi: 10.1093/annonc/mdx730 PMC5913595 29145561

[47] Garufi G Palazzo A Paris I Orlandi A Cassano A Tortora G. Neoadjuvant therapy for triple-negative breast cancer: potential predictive biomarkers of activity and efficacy of platinum chemotherapy, PARP- and immune-checkpoint-inhibitors. Expert Opin Pharmacother. (2020) 21:687–99. doi: 10.1080/14656566.2020.1724957 32052646

[48] Szöor A Tóth G Zsebik B Szabó V Eshhar Z Abken H. Trastuzumab derived HER2-specific CARs for the treatment of trastuzumab-resistant breast cancer: CAR T cells penetrate and eradicate tumors that are not accessible to antibodies. Cancer Lett. (2020) 484:1–8. doi: 10.1016/j.canlet.2020.04.008 32289441

[49] Ledys F Kalfeist L Galland L Limagne E Ladoire S. Therapeutic associations comprising anti-PD-1/PD-L1 in breast cancer: clinical challenges and perspectives. Cancers. (2021) 13:25. doi: 10.3390/cancers13235999 PMC8656936 34885109

[50] Curigliano G Locatelli M Fumagalli L Goldhirsch A. Immunizing against breast cancer: A new swing for an old sword. Breast. (2009) 18:S51–S4. doi: 10.1016/S0960-9776(09)70273-5 19914543

[51] Abdel-Aziz AK Saadeldin MK D’Amico P Orecchioni S Bertolini F Curigliano G. Preclinical models of breast cancer: Two-way shuttles for immune checkpoint inhibitors from and to patient bedside. Eur J Cancer. (2019) 122:22–41. doi: 10.1016/j.ejca.2019.08.013 31606656

[52] Esposito A Criscitiello C Curigliano G. Immune checkpoint inhibitors with radiotherapy and locoregional treatment: synergism and potential clinical implications. Curr Opin Oncol. (2015) 27:445–51. doi: 10.1097/CCO.0000000000000225 26447875

[53] Darwich A Silvestri A Benmebarek MR Mouriès J Cadilha B Melacarne A. Paralysis of the cytotoxic granule machinery is a new cancer immune evasion mechanism mediated by chitinase 3-like-1. J Immunother Cancer. (2021) 9:16. doi: 10.1136/jitc-2021-003224 PMC8627417 34824159

[54] Barroso-Sousa R Pacífico JP Sammons S Tolaney SM. Tumor mutational burden in breast cancer: current evidence, challenges, and opportunities. Cancers. (2023) 15:13. doi: 10.3390/cancers15153997 PMC10417019 37568813

[55] Goldberg J Pastorello RG Vallius T Davis J Cui YX Agudo J. The immunology of hormone receptor positive breast cancer. Front Immunol. (2021) 12:22. doi: 10.3389/fimmu.2021.674192 PMC8202289 34135901

[56] Ruiu R Tarone L Rolih V Barutello G Bolli E Riccardo F. Cancer stem cell immunology and immunotherapy: Harnessing the immune system against cancer’s source. In: Teplow DB, editor. Cancer Immunotherapy. Progress in Molecular Biology and Translational Science, vol. 164 . Elsevier Academic Press Inc, San Diego (2019). p. 119–88. 10.1016/bs.pmbts.2019.03.008 31383404

[57] Ruiu R Di Lorenzo A Cavallo F Conti L. Are cancer stem cells a suitable target for breast cancer immunotherapy? Front Oncol. (2022) 12:10. doi: 10.3389/fonc.2022.877384 PMC9069673 35530300

[58] Jiang P Gu SQ Pan D Fu JX Sahu A Hu XH. Signatures of T cell dysfunction and exclusion predict cancer immunotherapy response. Nat Med. (2018) 24:1550. doi: 10.1038/s41591-018-0136-1 30127393 PMC6487502

[59] Baldominos P Barbera-Mourelle A Barreiro O Huang Y Wight A Cho JW. Quiescent cancer cells resist T cell attack by forming an immunosuppressive niche. Cell. (2022) 185:1694. doi: 10.1016/j.cell.2022.03.033 35447074 PMC11332067

[60] Hanahan D Weinberg RA. Hallmarks of cancer: the next generation. Cell. (2011) 144:646–74. doi: 10.1016/j.cell.2011.02.013 21376230

[61] Khalaf K Hana D Chou JT Singh C Mackiewicz A Kaczmarek M. Aspects of the tumor microenvironment involved in immune resistance and drug resistance. Front Immunol. (2021) 12:656364. doi: 10.3389/fimmu.2021.656364 34122412 PMC8190405

[62] Kawaguchi K Maeshima Y Toi M. Tumor immune microenvironment and systemic response in breast cancer. Med Oncol. (2022) 39:208. doi: 10.1007/s12032-022-01782-0 36175677

[63] Sharma P Hu-Lieskovan S Wargo JA Ribas A. Primary, adaptive, and acquired resistance to cancer immunotherapy. Cell. (2017) 168:707–23. doi: 10.1016/j.cell.2017.01.017 PMC5391692 28187290

[64] Matkowski R Gisterek I Halon A Lacko A Szewczyk K Staszek U. The prognostic role of tumor-infiltrating CD4 and CD8 T lymphocytes in breast cancer. Anticancer Res. (2009) 29:2445–51. 19596912

[65] Kumar A Watkins R Vilgelm AE. Cell therapy with TILs: training and taming T cells to fight cancer. Front Immunol. (2021) 12:690499. doi: 10.3389/fimmu.2021.690499 34140957 PMC8204054

[66] Chen Y Klingen TA Aas H Wik E Akslen LA. Tumor-associated lymphocytes and macrophages are related to stromal elastosis and vascular invasion in breast cancer. J Pathol Clin Res. (2021) 7:517–27. doi: 10.1002/cjp2.226 PMC8363927 34076969

[67] Stanton SE Disis ML. Clinical significance of tumor-infiltrating lymphocytes in breast cancer. J Immunother Cancer. (2016) 4:59. doi: 10.1186/s40425-016-0165-6 27777769 PMC5067916

[68] Huang P Zhou X Zheng M Yu Y Jin G Zhang S. Regulatory T cells are associated with the tumor immune microenvironment and immunotherapy response in triple-negative breast cancer. Front Immunol. (2023) 14:1263537. doi: 10.3389/fimmu.2023.1263537 37767092 PMC10521732

[69] Scheinecker C Göschl L Bonelli M. Treg cells in health and autoimmune diseases: New insights from single cell analysis. J Autoimmun. (2020) 110:102376. doi: 10.1016/j.jaut.2019.102376 31862128

[70] Eggenhuizen PJ Ng BH Ooi JD. Treg enhancing therapies to treat autoimmune diseases. Int J Mol Sci. (2020) 21:944–61. doi: 10.3390/ijms21197015 PMC7582931 32977677

[71] Shan F Somasundaram A Bruno TC Workman CJ Vignali DAA. Therapeutic targeting of regulatory T cells in cancer. Trends Cancer. (2022) 8:944–61. doi: 10.1016/j.trecan.2022.06.008 PMC9588644 35853825

[72] Seif F Torki Z Zalpoor H Habibi M Pornour M. Breast cancer tumor microenvironment affects Treg/IL-17-producing Treg/Th17 cell axis: Molecular and therapeutic perspectives. Mol Ther Oncol. (2023) 28:132–57. doi: 10.1016/j.omto.2023.01.001 PMC9922830 36816749

[73] Korbecki J Grochans S Gutowska I Barczak K Baranowska-Bosiacka I. CC chemokines in a tumor: A review of pro-cancer and anti-cancer properties of receptors CCR5, CCR6, CCR7, CCR8, CCR9, and CCR10 ligands. Int J Mol Sci. (2020) 21:7619. doi: 10.3390/ijms21207619 33076281 PMC7590012

[74] Korbecki J Kojder K Simińska D Bohatyrewicz R Gutowska I Chlubek D. CC chemokines in a tumor: A review of pro-cancer and anti-cancer properties of the ligands of receptors CCR1, CCR2, CCR3, and CCR4. Int J Mol Sci. (2020) 21:8412. doi: 10.3390/ijms21218412 33182504 PMC7665155

[75] Tanaka A Sakaguchi S. Targeting Treg cells in cancer immunotherapy. Eur J Immunol. (2019) 49:1140–6. doi: 10.1002/eji.201847659 31257581

[76] Adams S Goldstein LJ Sparano JA Demaria S Badve SS. Tumor infiltrating lymphocytes (TILs) improve prognosis in patients with triple negative breast cancer (TNBC). OncoImmunology. (2015) 4:e985930. doi: 10.4161/2162402X.2014.985930 26405612 PMC4570112

[77] Palazón A Martínez-Forero I Teijeira A Morales-Kastresana A Alfaro C Sanmamed MF. The HIF-1α hypoxia response in tumor-infiltrating T lymphocytes induces functional CD137 (4-1BB) for immunotherapy. Cancer Discovery. (2012) 2:608–23. doi: 10.1158/2159-8290.CD-11-0314 22719018

[78] Clambey ET McNamee EN Westrich JA Glover LE Campbell EL Jedlicka P. Hypoxia-inducible factor-1 alpha-dependent induction of FoxP3 drives regulatory T-cell abundance and function during inflammatory hypoxia of the mucosa. Proc Natl Acad Sci U S A. (2012) 109:E2784–93. doi: 10.1073/pnas.1202366109 PMC3478644 22988108

[79] Ma SR Deng WW Liu JF Mao L Yu GT Bu LL. Blockade of adenosine A2A receptor enhances CD8(+) T cells response and decreases regulatory T cells in head and neck squamous cell carcinoma. Mol Cancer. (2017) 16:99. doi: 10.1186/s12943-017-0665-0 28592285 PMC5461710

[80] Chocarro L Blanco E Zuazo M Arasanz H Bocanegra A Fernández-Rubio L. Understanding LAG-3 signaling. Int J Mol Sci. (2021) 22:5282. doi: 10.3390/ijms22105282 34067904 PMC8156499

[81] Fu C Jiang A. Dendritic cells and CD8 T cell immunity in tumor microenvironment. Front Immunol. (2018) 9:3059. doi: 10.3389/fimmu.2018.03059 30619378 PMC6306491

[82] Gabrilovich D Ishida T Oyama T Ran S Kravtsov V Nadaf S. Vascular endothelial growth factor inhibits the development of dendritic cells and dramatically affects the differentiation of multiple hematopoietic lineages in vivo. Blood. (1998) 92:4150–66. doi: 10.1182/blood.V92.11.4150 9834220

[83] Kumar A Ramani V Bharti V Bellan DD Saleh N Uzhachenko R. Dendritic cell therapy augments antitumor immunity triggered by CDK4/6 inhibition and immune checkpoint blockade by unleashing systemic CD4 T-cell responses. J Immunother Cancer. (2023) 11:18. doi: 10.1136/jitc-2022-006019 PMC10231009 37230537

[84] Gautam N Ramamoorthi G Champion N Han HS Czerniecki BJ. Reviewing the significance of dendritic cell vaccines in interrupting breast cancer development. Mol Asp Med. (2024) 95:12. doi: 10.1016/j.mam.2023.101239 38150884

[85] Özverel CS Uyanikgil Y Karaboz I Nalbantsoy A. Investigation of the combination of anti-PD-L1 mAb with HER2/neu-loaded dendritic cells and QS-21 saponin adjuvant: effect against HER2 positive breast cancer in mice. Immunopharmacol Immunotoxicol. (2020) 42:346–57. doi: 10.1080/08923973.2020.1775644 32515626

[86] Xu YN Zheng B Huang MQ Li XH Wang ZY Chang J. Sendai virus acts as a nano-booster to excite dendritic cells for enhancing the efficacy of CD47-directed immune checkpoint inhibitors against breast carcinoma. Mat Chem Front. (2021) 5:223–37. doi: 10.1039/D0QM00393J

[87] Feng B Wu JZ Shen B Jiang F Feng JF. Cancer-associated fibroblasts and resistance to anticancer therapies: status, mechanisms, and countermeasures. Cancer Cell Int. (2022) 22:15. doi: 10.1186/s12935-022-02599-7 35488263 PMC9052457

[88] Zhu SX Wang YM Tang J Cao M. Radiotherapy induced immunogenic cell death by remodeling tumor immune microenvironment. Front Immunol. (2022) 13:20. doi: 10.3389/fimmu.2022.1074477 PMC9753984 36532071

[89] Valenti G Quinn HM Heynen G Lan L Holland JD Vogel R. Cancer stem cells regulate cancer-associated fibroblasts via activation of hedgehog signaling in mammary gland tumors. Cancer Res. (2017) 77:2134–47. doi: 10.1158/0008-5472.CAN-15-3490 28202523

[90] Magagna I Gourdin N Kieffer Y Licaj M Mhaidly R Andre P. CD73-mediated immunosuppression is linked to a specific fibroblast population that paves the way for new therapy in breast cancer. Cancers. (2021) 13:21. doi: 10.3390/cancers13235878 PMC8657241 34884993

[91] Huelsken J Hanahan D. A subset of cancer-associated fibroblasts determines therapy resistance. Cell. (2018) 172:643–4. doi: 10.1016/j.cell.2018.01.028 29425485

[92] Costa A Kieffer Y Scholer-Dahirel A Pelon F Bourachot B Cardon M. Fibroblast heterogeneity and immunosuppressive environment in human breast cancer. Cancer Cell. (2018) 33:463–79.e10. doi: 10.1016/j.ccell.2018.01.011 29455927

[93] Akalay I Janji B Hasmim M Noman MZ André F De Cremoux P. Epithelial-to-mesenchymal transition and autophagy induction in breast carcinoma promote escape from T-cell-mediated lysis. Cancer Res. (2013) 73:2418–27. doi: 10.1158/0008-5472.CAN-12-2432 23436798

[94] Rahir G Moser M. Tumor microenvironment and lymphocyte infiltration. Cancer Immunol Immunother. (2012) 61:751–9. doi: 10.1007/s00262-012-1253-1 PMC11028584 22488275

[95] Allen E Jabouille A Rivera LB Lodewijckx I Missiaen R Steri V. Combined antiangiogenic and anti-PD-L1 therapy stimulates tumor immunity through HEV formation. Sci Transl Med. (2017) 9:13. doi: 10.1126/scitranslmed.aak9679 PMC5554432 28404866

[96] Tokunaga R Zhang W Naseem M Puccini A Berger MD Soni S. CXCL9, CXCL10, CXCL11/CXCR3 axis for immune activation - A target for novel cancer therapy. Cancer Treat Rev. (2018) 63:40–7. doi: 10.1016/j.ctrv.2017.11.007 PMC5801162 29207310

[97] Hegde PS Chen DS. Top 10 challenges in cancer immunotherapy. Immunity. (2020) 52:17–35. doi: 10.1016/j.immuni.2019.12.011 31940268

[98] Fang Y Wang L Wan C Sun Y van der Jeught K Zhou Z. MAL2 drives immune evasion in breast cancer by suppressing tumor antigen presentation. J Clin Invest. (2021) 131:e140837. doi: 10.1172/JCI140837 32990678 PMC7773365

[99] Xu QC Liu XY Mohseni G Hao XD Ren YD Xu YW. Mechanism research and treatment progress of NAD pathway related molecules in tumor immune microenvironment. Cancer Cell Int. (2022) 22:21. doi: 10.1186/s12935-022-02664-1 35906622 PMC9338646

[100] Pardoll DM. The blockade of immune checkpoints in cancer immunotherapy. Nat Rev Cancer. (2012) 12:252–64. doi: 10.1038/nrc3239 PMC4856023 22437870

[101] Kern R Panis C. CTLA-4 expression and its clinical significance in breast cancer. Arch Immunol Ther Exp. (2021) 69:9. doi: 10.1007/s00005-021-00618-5 34148159

[102] Tumeh PC Harview CL Yearley JH Shintaku IP Taylor EJ Robert L. PD-1 blockade induces responses by inhibiting adaptive immune resistance. Nature. (2014) 515:568–71. doi: 10.1038/nature13954 PMC4246418 25428505

[103] Schmid P Adams S Rugo HS Schneeweiss A Barrios CH Iwata H. Atezolizumab and nab-paclitaxel in advanced triple-negative breast cancer. N Engl J Med. (2018) 379:2108–21. doi: 10.1056/NEJMoa1809615 30345906

[104] Yi KH Chen L. Fine tuning the immune response through B7-H3 and B7-H4. Immunol Rev. (2009) 229:145–51. doi: 10.1111/j.1600-065X.2009.00768.x PMC2696224 19426220

[105] Sisinni L Pietrafesa M Lepore S Maddalena F Condelli V Esposito F. Endoplasmic reticulum stress and unfolded protein response in breast cancer: the balance between apoptosis and autophagy and its role in drug resistance. Int J Mol Sci. (2019) 20:857. doi: 10.3390/ijms20040857 30781465 PMC6412864

[106] Cubillos-Ruiz JR Bettigole SE Glimcher LH. Tumorigenic and immunosuppressive effects of endoplasmic reticulum stress in cancer. Cell. (2017) 168:692–706. doi: 10.1016/j.cell.2016.12.004 28187289 PMC5333759

[107] Li J Zhang Y Cai YF Yao PZ Jia YW Wei XY. Multi-omics analysis elucidates the relationship between intratumor microbiome and host immune heterogeneity in breast cancer. Microbiol Spectr. (2024) 20:e0410423. doi: 10.1128/spectrum.04104-23 PMC10986513 38442004

[108] Arner EN Rathmell JC. Metabolic programming and immune suppression in the tumor microenvironment. Cancer Cell. (2023) 41:421–33. doi: 10.1016/j.ccell.2023.01.009 PMC10023409 36801000

[109] Cham CM Driessens G O’Keefe JP Gajewski TF. Glucose deprivation inhibits multiple key gene expression events and effector functions in CD8+ T cells. Eur J Immunol. (2008) 38:2438–50. doi: 10.1002/eji.200838289 PMC3008428 18792400

[110] Naik A Decock J. Lactate metabolism and immune modulation in breast cancer: A focused review on triple negative breast tumors. Front Oncol. (2020) 10:598626. doi: 10.3389/fonc.2020.598626 33324565 PMC7725706

[111] Tang YH Tian WW Xie JD Zou YT Wang ZH Li N. Prognosis and dissection of immunosuppressive microenvironment in breast cancer based on fatty acid metabolism-related signature. Front Immunol. (2022) 13:17. doi: 10.3389/fimmu.2022.843515 PMC9009264 35432381

[112] Ogretmen B. Sphingolipid metabolism in cancer signalling and therapy. Nat Rev Cancer. (2018) 18:33–50. doi: 10.1038/nrc.2017.96 29147025 PMC5818153

[113] Luo YZ Tian WW Lu XQ Zhang C Xie JD Deng XP. Prognosis stratification in breast cancer and characterization of immunosuppressive microenvironment through a pyrimidine metabolism-related signature. Front Immunol. (2022) 13:19. doi: 10.3389/fimmu.2022.1056680 PMC9745154 36524129

[114] Yang F Yang YQ Qiu YL Tang L Xie L Guan XX. Long non-coding RNAs as regulators for targeting breast cancer stem cells and tumor immune microenvironment: biological properties and therapeutic potential. Cancers. (2024) 16:15. doi: 10.3390/cancers16020290 PMC10814583 38254782

[115] Li Y Chen Z Wu L Ye J Tao W. Cellular heterogeneity map of diverse immune and stromal phenotypes within breast tumor microenvironment. PeerJ. (2020) 8:e9478. doi: 10.7717/peerj.9478 32728493 PMC7357563

[116] Wang ZQ Milne K Derocher H Webb JR Nelson BH Watson PH. PD-L1 and intratumoral immune response in breast cancer. Oncotarget. (2017) 8:51641–51. doi: 10.18632/oncotarget.v8i31 PMC5584276 28881675

[117] Núñez Abad M Calabuig-Fariñas S Lobo de Mena M Torres-Martínez S García González C García García J. Programmed death-ligand 1 (PD-L1) as immunotherapy biomarker in breast cancer. Cancers (Basel). (2022) 14:307. doi: 10.3390/cancers14020307 35053471 PMC8773553

[118] Tomioka N Azuma M Ikarashi M Yamamoto M Sato M Watanabe KI. The therapeutic candidate for immune checkpoint inhibitors elucidated by the status of tumor-infiltrating lymphocytes (TILs) and programmed death ligand 1 (PD-L1) expression in triple negative breast cancer (TNBC). Breast Cancer. (2018) 25:34–42. doi: 10.1007/s12282-017-0781-0 28488168

[119] Rizzo A Ricci AD. Biomarkers for breast cancer immunotherapy: PD-L1, TILs, and beyond. Expert Opin Investig Drugs. (2022) 31:549–55. doi: 10.1080/13543784.2022.2008354 34793275

[120] Tabana Y Okoye IS Siraki A Elahi S Barakat KH. Tackling immune targets for breast cancer: beyond PD-1/PD-L1 axis. Front Oncol. (2021) 11:628138. doi: 10.3389/fonc.2021.628138 33747948 PMC7973280

[121] Kanwar N Balde Z Nair R Dawe M Chen S Maganti M. Heterogeneity of circulating tumor cell-associated genomic gains in breast cancer and its association with the host immune response. Cancer Res. (2021) 81:6196–206. doi: 10.1158/0008-5472.CAN-21-1079 PMC9397625 34711609

[122] Sukumar J Gast K Quiroga D Lustberg M Williams N. Triple-negative breast cancer: promising prognostic biomarkers currently in development. Expert Rev Anticancer Ther. (2021) 21:135–48. doi: 10.1080/14737140.2021.1840984 PMC8174647 33198517

[123] Xu Q Chen S Hu Y Huang W. Landscape of immune microenvironment under immune cell infiltration pattern in breast cancer. Front Immunol. (2021) 12:711433. doi: 10.3389/fimmu.2021.711433 34512634 PMC8429934

[124] Sammons S Elliott A Barroso-Sousa R Chumsri S Tan AR Sledge GW Jr.. Concurrent predictors of an immune responsive tumor microenvironment within tumor mutational burden-high breast cancer. Front Oncol. (2023) 13:1235902. doi: 10.3389/fonc.2023.1235902 37637072 PMC10457522

[125] Bergholz JS Wang Q Wang Q Ramseier M Prakadan S Wang W. PI3Kβ controls immune evasion in PTEN-deficient breast tumours. Nature. (2023) 617:139–46. doi: 10.1038/s41586-023-05940-w PMC10494520 37076617

[126] Blenman KRM Marczyk M Karn T Qing T Li X Gunasekharan V. Predictive markers of response to neoadjuvant durvalumab with nab-paclitaxel and dose-dense doxorubicin/cyclophosphamide in basal-like triple-negative breast cancer. Clin Cancer Res. (2022) 28:2587–97. doi: 10.1158/1078-0432.CCR-21-3215 PMC9464605 35377948

[127] Proietto M Crippa M Damiani C Pasquale V Sacco E Vanoni M. Tumor heterogeneity: preclinical models, emerging technologies, and future applications. Front Oncol. (2023) 13:24. doi: 10.3389/fonc.2023.1164535 PMC10175698 37188201

[128] Yu LY Tang J Zhang CM Zeng WJ Yan H Li MP. New immunotherapy strategies in breast cancer. Int J Environ Res Public Health. (2017) 14:68. doi: 10.3390/ijerph14010068 28085094 PMC5295319

[129] Sanchez K Page D McArthur HL. Immunotherapy in breast cancer: An overview of modern checkpoint blockade strategies and vaccines. Curr Probl Cancer. (2016) 40:151–62. doi: 10.1016/j.currproblcancer.2016.09.009 27855963

[130] Guerrouahen BS Maccalli C Cugno C Rutella S Akporiaye ET. Reverting immune suppression to enhance cancer immunotherapy. Front Oncol. (2019) 9:1554. doi: 10.3389/fonc.2019.01554 32039024 PMC6985581

[131] Hartmann L Osen W Eichmüller OL Kordass T Furkel J Dickes E. Carbon ion irradiation plus CTLA4 blockade elicits therapeutic immune responses in a murine tumor model. Cancer Lett. (2022) 550:16. doi: 10.1016/j.canlet.2022.215928 36183858

[132] Cortes J Rugo HS Cescon DW Im SA Yusof MM Gallardo C. Pembrolizumab plus chemotherapy in advanced triple-negative breast cancer. N Engl J Med. (2022) 387:217–26. doi: 10.1056/NEJMoa2202809 35857659

[133] Ye F Dewanjee S Li Y Jha NK Chen ZS Kumar A. Advancements in clinical aspects of targeted therapy and immunotherapy in breast cancer. Mol Cancer. (2023) 22:105. doi: 10.1186/s12943-023-01805-y 37415164 PMC10324146

[134] Pack CD Bommireddy R Munoz LE Patel JM Bozeman EN Dey P. Tumor membrane-based vaccine immunotherapy in combination with anti-CTLA-4 antibody confers protection against immune checkpoint resistant murine triple-negative breast cancer. Hum Vaccin Immunother. (2020) 16:3184–93. doi: 10.1080/21645515.2020.1754691 PMC8641616 32530786

[135] Emens LA. Breast cancer immunotherapy: facts and hopes. Clin Cancer Res. (2018) 24:511–20. doi: 10.1158/1078-0432.CCR-16-3001 PMC5796849 28801472

[136] Lee YJ Auh SL Wang YG Burnette B Wang Y Meng YR. Therapeutic effects of ablative radiation on local tumor require CD8+ T cells: changing strategies for cancer treatment. Blood. (2009) 114:589–95. doi: 10.1182/blood-2009-02-206870 PMC2713472 19349616

[137] Zhang SM Xu QM Sun WJ Zhou JY Zhou JY. Immunomodulatory effects of CDK4/6 inhibitors. Biochim Biophys Acta-Rev Cancer. (2023) 1878:11. doi: 10.1016/j.bbcan.2023.188912 37182667

[138] Zhang ZZ Richmond A. The role of PI3K inhibition in the treatment of breast cancer, alone or combined with immune checkpoint inhibitors. Front Mol Biosci. (2021) 8:10. doi: 10.3389/fmolb.2021.648663 PMC8139556 34026830

[139] Mariathasan S Turley SJ Nickles D Castiglioni A Yuen K Wang Y. TGFβ attenuates tumour response to PD-L1 blockade by contributing to exclusion of T cells. Nature. (2018) 554:544–8. doi: 10.1038/nature25501 PMC6028240 29443960

[140] Yan L Wu M Wang T Yuan H Zhang X Zhang H. Breast cancer stem cells secrete MIF to mediate tumor metabolic reprogramming that drives immune evasion. Cancer Res. (2024) 84:1270–85. doi: 10.1158/0008-5472.CAN-23-2390 38335272

[141] Nasiri F Kazemi M Mirarefin SMJ Mahboubi Kancha M Ahmadi Najafabadi M Salem F. CAR-T cell therapy in triple-negative breast cancer: Hunting the invisible devil. Front Immunol. (2022) 13:1018786. doi: 10.3389/fimmu.2022.1018786 36483567 PMC9722775

[142] Iizuka A Nonomura C Ashizawa T Kondou R Ohshima K Sugino T. A T-cell-engaging B7-H4/CD3-bispecific fab-scFv antibody targets human breast cancer. Clin Cancer Res. (2019) 25:2925–34. doi: 10.1158/1078-0432.CCR-17-3123 30737243

[143] Klemm F Joyce JA. Microenvironmental regulation of therapeutic response in cancer. Trends Cell Biol. (2015) 25:198–213. doi: 10.1016/j.tcb.2014.11.006 25540894 PMC5424264

[144] Kim PS Armstrong TD Song H Wolpoe ME Weiss V Manning EA. Antibody association with HER-2/neu-targeted vaccine enhances CD8 T cell responses in mice through Fc-mediated activation of DCs. J Clin Invest. (2008) 118:1700–11. doi: 10.1172/JCI34333 PMC2289797 18398507

[145] Nilsson N Carlsten H. Estrogen induces suppression of natural killer cell cytotoxicity and augmentation of polyclonal B cell activation. Cell Immunol. (1994) 158:131–9. doi: 10.1006/cimm.1994.1262 8087860

[146] Huang HH Zhou J Chen HL Li JX Zhang C Jiang X. The immunomodulatory effects of endocrine therapy in breast cancer. J Exp Clin Cancer Res. (2021) 40:16. doi: 10.1186/s13046-020-01788-4 33413549 PMC7792133

[147] Tomita Y Lee MJ Lee S Tomita S Chumsri S Cruickshank S. The interplay of epigenetic therapy and immunity in locally recurrent or metastatic estrogen receptor-positive breast cancer: Correlative analysis of ENCORE 301, a randomized, placebo-controlled phase II trial of exemestane with or without entinostat. OncoImmunology. (2016) 5:e1219008. doi: 10.1080/2162402X.2016.1219008 27999738 PMC5139687

[148] Lv T Yuan D Miao X Lv Y Zhan P Shen X. Over-expression of LSD1 promotes proliferation, migration and invasion in non-small cell lung cancer. PloS One. (2012) 7:e35065. doi: 10.1371/journal.pone.0035065 22493729 PMC3320866

[149] Braun TP Coblentz C Curtiss BM Coleman DJ Schonrock Z Carratt SA. Combined inhibition of JAK/STAT pathway and lysine-specific demethylase 1 as a therapeutic strategy in CSF3R/CEBPA mutant acute myeloid leukemia. Proc Natl Acad Sci U S A. (2020) 117:13670–9. doi: 10.1073/pnas.1918307117 PMC7306806 32471953

[150] Lee DY Salahuddin T Iqbal J. Lysine-specific demethylase 1 (LSD1)-mediated epigenetic modification of immunogenicity and immunomodulatory effects in breast cancers. Curr Oncol. (2023) 30:2127–43. doi: 10.3390/curroncol30020164 PMC9955398 36826125

[151] Hosseini M Haji-Fatahaliha M Jadidi-Niaragh F Majidi J Yousefi M. The use of nanoparticles as a promising therapeutic approach in cancer immunotherapy. Artif Cell Nanomed Biotechnol. (2016) 44:1051–61. doi: 10.3109/21691401.2014.998830 25612903

[152] Shanavas A Jain NK Kaur N Thummuri D Prasanna M Prasad R. Polymeric core-shell combinatorial nanomedicine for synergistic anticancer therapy. ACS Omega. (2019) 4:19614–22. doi: 10.1021/acsomega.9b02167 PMC6881840 31788591

[153] Kommineni N Chaudhari R Conde J Tamburaci S Cecen B Chandra P. Engineered liposomes in interventional theranostics of solid tumors. ACS Biomater Sci Eng. (2023) 9:4527–57. doi: 10.1021/acsbiomaterials.3c00510 37450683

[154] Chauhan DS Dhasmana A Laskar P Prasad R Jain NK Srivastava R. Nanotechnology synergized immunoengineering for cancer. Eur J Pharm Biopharm. (2021) 163:72–101. doi: 10.1016/j.ejpb.2021.03.010 33774162 PMC8170847

[155] Qian C Yang LJ Cui H. Recent advances in nanotechnology for dendritic cell-based immunotherapy. Front Pharmacol. (2020) 11:9. doi: 10.3389/fphar.2020.00960 32694998 PMC7338589

[156] Kumari N Choi SH. Tumor-associated macrophages in cancer: recent advancements in cancer nanoimmunotherapies. J Exp Clin Cancer Res. (2022) 41:39. doi: 10.1186/s13046-022-02272-x 35183252 PMC8857848

[157] Huang J Xiao ZC Chen GJ Li T Peng Y Shuai XT. A pH-sensitive nanomedicine incorporating catalase gene and photosensitizer augments photodynamic therapy and activates antitumor immunity. Nano Today. (2022) 43:11. doi: 10.1016/j.nantod.2022.101390

[158] Shukla S Jandzinski M Wang C Gong XJ Bonk KW Keri RA. A viral nanoparticle cancer vaccine delays tumor progression and prolongs survival in a HER2+ Tumor mouse model. Adv Therap. (2019) 2:11. doi: 10.1002/adtp.201800139 PMC8043622 33855164

